# Anticancer Properties of Selected Wood-Inhabiting Fungi

**DOI:** 10.3390/nu18152495

**Published:** 2026-08-02

**Authors:** Piotr Roszczenko, Olga Klaudia Szewczyk-Roszczenko, Agnieszka Gornowicz, Robert Czarnomysy, Yegor Vassetzky, Krzysztof Bielawski, Monika Wujec, Anna Bielawska

**Affiliations:** 1 Department of Biotechnology, Medical University of Białystok, Kilinskiego 1, 15-089 Bialystok, Poland; 2 Department of Synthesis and Technology of Drugs, Medical University of Bialystok, Kilinskiego 1, 15-089 Bialystok, Poland; 3 Chromatin Dynamics in Cancer, CNRS UMR9018 Institut Gustave Roussy, Univeristé Paris Saclay, 39 Rue Camille-Desmoulins, 94805 Villejuif, France; 4 Department of Organic Chemistry, Faculty of Pharmacy, Medical University of Lublin, 4A Chodzki Street, 20-093 Lublin, Poland

**Keywords:** tree fungi, anticancer treatment, bioactive compounds, mushroom extracts, fungal metabolites, cancer therapy

## Abstract

Wood-inhabiting fungi constitute a valuable source of biologically active compounds with significant therapeutic potential. Particular attention is paid to species known for their high content of polysaccharides, triterpenoids, phenolic compounds, and other secondary metabolites exhibiting cytotoxic and immunomodulatory activities. The results indicate that extracts obtained from these fungi may inhibit tumor growth, induce apoptosis, and support the immune response of the organism. Although further clinical studies are required, wood-inhabiting medicinal fungi represent a promising direction in the development of natural anticancer therapies and complementary treatment strategies.

## 1. Introduction

The shared evolutionary origin of fungi and animals is one of the key factors explaining the biological activity of fungal metabolites against mammalian cells. Both groups of organisms belong to the *Opisthokonta* supergroup, which means they are descended from a common ancestor that lived around a billion years ago. Despite significant morphological and physiological differences, fungi and animals have retained many similar molecular mechanisms regulating basic cellular processes. These include, amongst others, DNA replication, gene expression, chromatin organization, the cell cycle, responses to oxidative stress, and DNA damage repair systems [1]. The high degree of conservation of these pathways means that chemical compounds acting on fungal cells may also affect analogous structures and processes in mammalian cells [2].

It is particularly significant that many proteins performing key cellular functions share a similar structure and mechanism of action in both fungi and animals. This applies, for example, to DNA polymerases, histones, protein kinases and enzymes involved in regulating oxidative balance. Consequently, secondary metabolites produced by fungi may interact with molecular targets also present in mammalian cells. Although these compounds did not evolve specifically to affect human organisms, due to preserved evolutionary similarities, they may modulate the same biological pathways in various eukaryotic organisms. This is precisely why many fungal metabolites exhibit genotoxic, cytotoxic or regulatory activity in human cell models [3].

This shared evolutionary origin also accounts for the similarity in cellular response mechanisms to stress and DNA damage. Both fungi and mammalian cells employ conserved systems for detecting damage to genetic material and activating repair processes. Fungal metabolites can therefore interfere with the same signaling pathways responsible for maintaining genomic stability. In some cases, this leads to the accumulation of mutations or oxidative stress, whilst in others it may activate protective and adaptive mechanisms [4].

From an evolutionary perspective, fungal metabolites can therefore be viewed as chemical compounds that interact with universal elements of eukaryotic cell biology. Their activity towards mammalian organisms is not random, but stems from deeply rooted molecular similarities preserved since the time of the common ancestor of fungi and animals.

The molecular similarities between fungal and animal cells have made fungal metabolites the subject of extensive research into their potential anticancer effects. However, most of the available evidence is derived from in vitro studies using cancer cell lines, where biological activity is primarily evaluated through cell viability assays such as MTT or XTT. Although these models remain indispensable for the initial screening of bioactive compounds, they fail to fully reproduce the complexity of the tumor microenvironment and the dynamic interactions between different cell types. To overcome these limitations, more advanced experimental platforms, including organ-on-a-chip (OoC) systems, have recently been developed. By integrating microfluidic technology with three-dimensional cell cultures, OoC models more closely mimic the physiological architecture and function of human tissues and are increasingly being applied in disease modeling, drug discovery, and the evaluation of natural products. Although their application in studies of anticancer metabolites derived from wood-inhabiting fungi remains limited, these systems represent a promising approach that could complement conventional in vitro assays and improve the predictive value of preclinical efficacy and safety assessments [5].

Despite the promising anticancer activity demonstrated by numerous metabolites isolated from wood-inhabiting fungi, their toxicological characterization remains incomplete. Most available studies are limited to in vitro cytotoxicity assays, whereas comprehensive in vivo toxicity studies, pharmacokinetic (ADME) investigations, dose–response analyses, and long-term safety evaluations are largely lacking. Consequently, the safety range and therapeutic window of most fungal-derived metabolites have not yet been established, representing a major challenge for their further preclinical and clinical development [6].

The biological activity and applicability of fungal products, particularly polysaccharides, are closely related to their structural characteristics, including molecular weight, monosaccharide composition, and the presence of a β-(1 → 3)-glucan backbone with β-(1 → 6) branches, which is associated with the strongest immunomodulatory and antitumor activities. Chemical modifications of these polysaccharides, which improve their solubility and alter their physicochemical properties, can further enhance their biological activity, highlighting the strong structure–activity relationship [7].

The available evidence indicates that the anticancer activity of metabolites isolated from wood-inhabiting fungi is mediated by a combination of direct cytotoxic mechanisms, including apoptosis induction, cell cycle arrest, DNA damage, autophagy, and inhibition of cancer cell migration, together with indirect immunomodulatory effects that enhance antitumor immune responses [8].

## 2. Structure–Activity Relationships of Major Anticancer Metabolites

Lanostane-type triterpenoids, particularly ganoderic acids, represent the major low-molecular-weight bioactive substances. Their anti-cancer potency is strongly influenced by the number and position of hydroxyl, carbonyl, and carboxyl groups, which modulate their cytotoxicity and ability to regulate apoptosis, cell cycle progression, angiogenesis, and metastatic signaling pathways. Several ganoderic acids have been shown to inhibit cancer cell proliferation through suppression of the PI3K/Akt, NF-κB, and MAPK pathways while promoting caspase-dependent apoptosis [9,10].

In contrast, the anti-cancer activity of polysaccharides is largely determined by their molecular weight, degree of branching, monosaccharide composition, and three-dimensional conformation. β-(1 → 3)-D-glucan backbones with β-(1 → 6)-linked side chains and triple-helix conformations exhibit the strongest immunomodulatory and antitumor properties, whereas structural modifications such as sulfation or carboxymethylation may further improve water solubility and biological activity [11]. These structural features facilitate the recognition of polysaccharides by pattern-recognition receptors, including Dectin-1 and Toll-like receptors, leading to macrophage and dendritic cell activation, cytokine production, and enhanced antitumor immune responses. In addition to indirect immunomodulation, polysaccharides can inhibit tumor growth by inducing apoptosis, suppressing angiogenesis, and reducing metastatic potential [12,13]. Recent evidence suggests that gut microbiota is an important mediator of the biological activity of fungal polysaccharides. By interacting with the intestinal mucus layer and modulating gut microbial composition and metabolism, fungal polysaccharides may enhance intestinal and systemic immune responses, thereby contributing to their anti-cancer effects [14,15]. Additionally, recent preclinical studies suggest that polysaccharides may enhance the efficacy of immune checkpoint inhibitors. In murine models, the combination of *G. lucidum* polysaccharides or sporoderm-removed *G. lucidum* spore powder with anti-PD-1/PD-L1 therapy significantly improved anti-tumor efficacy by increasing CD8+ T-cell infiltration, promoting Th1 immune responses, and reducing immunosuppressive cell populations compared with immune checkpoint blockade alone [16,17].

Besides triterpenoids and polysaccharides, phenolic compounds, sterols, proteins, and other secondary metabolites can contribute to anti-cancer activity. Phenolic compounds primarily exert antioxidant effects by scavenging reactive oxygen species, thereby protecting cells from oxidative damage, while also contributing to the modulation of cancer-related signaling pathways. Although their individual cytotoxic effects are generally weaker than those of triterpenoids, increasing evidence suggests that these compounds act synergistically with polysaccharides and triterpenoids, enhancing the overall therapeutic potential [18].

## 3. Order *Polyporales*

### 3.1. Family Polyporaceae—Cerrena unicolor

*Cerrena unicolor* is a white rot fungus also known as “Turkey Tail” or “Mossy Maze Polypore”. It is widespread in the environment, especially in the deciduous and mixed forests of Europe and North America. This species is primarily a highly efficient producer of laccase, an extracellularly secreted enzyme. The anticancer properties of *C. unicolor* are mainly associated with the presence of this enzyme (Table 1) [19].

Laccase (LAC) belongs to the group of blue multi-copper oxidases and is characterized by its unique redox capacity of copper ions. It catalyzes the oxidation of a wide range of aromatic compounds while reducing molecular oxygen to water. This enzyme is commonly found in plants, fungi, and bacteria. Its diverse biological properties make it useful in many fields. Laccase has long been known for its numerous biological activities, including its ability to effectively break down lignin, the main component of wood. This ability is also associated with the inhibition of the proliferation of certain cancer cells. Therefore, laccase is considered a promising candidate for biomedical applications [20,21].

Three bioactive fractions were isolated from idiomorphic cultures of *C. unicolor*: laccase (LAC), crude polysaccharides (c-EPL), and a low-molecular-weight fraction of secondary metabolites (ex-LMS; <10 kDa). All fractions exhibited antioxidant, antibacterial, antiviral, and immunomodulatory activities. Anticancer properties were observed for LAC and ex-LMS but not for c-EPL [22]. The ex-LMS fraction was further divided into three subfractions. Ex-LMS I included compounds with a molecular weight below 700 Da (12.25 µg/mL of proteins, 36.82 µg/mL of carbohydrates). Ex-LMS II contained molecules in the range of 700 Da–10 kDa (4.87 µg/mL of proteins, 29.35 µg/mL of carbohydrates). Ex-LMS III was a low-molecular-weight protein fraction (43.7 µg/mL of proteins, 5.48 µg/mL of carbohydrates) [23].

### 3.2. Family Polyporaceae—Daedaleopsis confragosa

Daedaleopsis confragosa, also known as “the blushing bracket”, is a common arboreal fungus found almost year-round [24]. It mainly inhabits deciduous trees such as willow, alder, and birch. It causes white rot, a process in which lignin is decomposed while light-colored cellulose is retained [25]. In addition to its important role in forest ecosystems, this species also exhibits interesting biological properties. Extracts from *D. confragosa* are characterized by antimicrobial, antioxidant and protective effects against genetic material [24].

Various types of extracts were analyzed in studies on the anticancer activity of *Daedaleopsis confragosa* (Table 2). Crude methanol extract from the fruiting body showed an inhibitory effect on the growth of cancer cells in vitro [26]. Water extracts of strain F-1368, in two variants K-3 (polysaccharide to protein ratio 5.8) and K-4 (1.4), showed significant activity both in vitro and in vivo [27]. Ethanol extracts were also studied: DC (obtained by conventional methods) and DCHD (obtained after hydrodistillation). These extracts differed in their content of phenolic and flavonoid compounds [25]. The results concerning the anticancer activity of these extracts are presented below.

### 3.3. Family Polyporaceae—Fomes fomentarius

**Figure 1:**
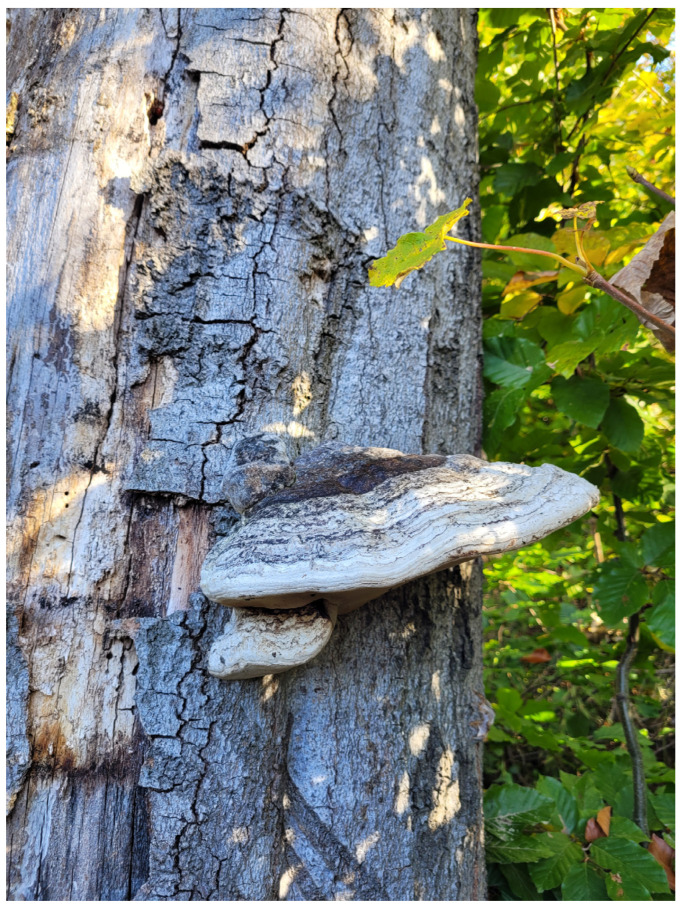
*Fomes fomentarius* found in its natural habitat in the Roztocze region, Poland.

*Fomes fomentarius* and the bioactive compounds contain a wide spectrum of biological activity. These include antibacterial, anticancer, antidiabetic, anti-inflammatory, antioxidant, analgesic, and hypoglycemic properties. Tinder conk, an inedible and cosmopolitan species known in Chinese as “Mudi,” forms large, woody fruiting bodies that develop as saprotrophs on deciduous trees (Figure 1). Used for centuries in traditional Chinese and Korean medicine to treat, among other things, mouth ulcers, gastrointestinal disorders, cirrhosis of the liver, inflammation, and cancer, this species is well known in ethnomedicine. It is currently the subject of intensive research aimed at confirming and explaining its potential anticancer properties [28,29,30].

A wide range of extracts and derivatives were used in studies on the anticancer activity of *Fomes fomentarius*. Cyclohexane, dichloromethane, methanol, and water extracts obtained from the fruiting bodies of the fungus were evaluated for their activity against various cancerous and normal cell lines [31]. In addition, aqueous, methanol, and ethanol extracts were analyzed against MCF-7 breast cancer cells and their chemotherapy-resistant variants [29]. Ethanol extract from fruiting bodies and from mycelium obtained in submerged culture were also evaluated [32]. Using the intracellular mushroom extract, silver nanoparticles (AgNPs) and titanium oxide nanoparticles (TiO_2_NPs) were synthesized and then tested for their cytotoxicity against cancer cells [33]. The activity of several polysaccharide products was also evaluated. These included exopolysaccharide (EPS) [28], intracellular polysaccharide (IPS) [32], and a water-soluble polysaccharide isolated from fruiting bodies (MFKF-AP1b) [34]. The results of the studies are summarized in the table below (Table 3), which compares the anticancer activity of all the fractions described.

### 3.4. Family Polyporaceae—Trametes versicolor

*Trametes versicolor* also known as *Coriolus versicolor*, *Polyporus versicolor*, Yun-Zhi, kawaratake, or Turkey tail mushroom, belongs to the lignicolous fungi that cause white rot. It is commonly found in the temperate zones of Asia, North America, and Europe. It colonizes various species of deciduous trees, including oak and plum, as well as conifers such as fir and pine. Although it is an inedible species, it has been used in traditional Chinese medicine for centuries. Its biological properties include antioxidant, anti-inflammatory, immunomodulatory, and anticancer effects. Numerous studies and clinical trials confirm its anticancer activity, resulting both from the direct cytotoxic effect of bioactive compounds and from the regulation of the immune response (Table 4). Of particular importance here is polysaccharide K (PSK, krestin). It has been approved by the Japanese National Health Registry as an anticancer drug since 1977 and is routinely used in cancer therapy in Japan [36,37,38].

Polysaccharopeptide (PSP) from *Trametes versicolor* exhibits potent immunomodulatory properties, primarily by stimulating a pro-inflammatory response and activating both innate and adaptive immunity. In vitro and in vivo studies have shown that PSP increases the production of cytokines such as TNF-α, IL-1β, IL-6, IL-12 and IFN-γ. This leads to the activation of macrophages, NK cells and T lymphocytes. This compound also enhances lymphocyte proliferation, macrophage phagocytic activity and antibody production. The mechanism of action of PSP is mainly associated with the presence of β-glucans, which interact with Dectin-1 and TLR2/TLR4 receptors. This interaction activates signaling pathways related to the immune response. PSP also exhibits potential anticancer activity. It inhibits the proliferation of cancer cells, inducing apoptosis, and increasing the sensitivity of cells to chemotherapy. These effects were observed particularly in HL-60 leukemia cell lines. At the same time, it is emphasized that the action of PSP is pleiotropic—some of the induced cytokines may, under certain conditions, promote immunosuppression or tumor progression [39].

PSK (Polysaccharide K, Krestin^®^), the second major polysaccharide-protein complex isolated from *Trametes versicolor*, also exhibits similar immunomodulatory properties. PSK contains protein-bound β-glucans and has been used in Japan since the 1970s as an adjuvant in cancer therapy, particularly for stomach, colorectal and lung cancer. The mechanism of action of PSK is primarily based on the modulation of the immune response through the activation of T lymphocytes, NK cells, macrophages and dendritic cells. It also regulates the production of cytokines such as IL-2, IFN-γ and TNF-α. It has also been demonstrated that PSK can limit the action of immunosuppressive factors present in the tumor microenvironment, including TGF-β and IL-10, restoring the balance of the Th1/Th2 response in favor of antitumor immunity. In addition to its effects on the immune system, PSK also exhibits direct anticancer activity. It inhibits tumor cell proliferation, induces apoptosis, suppresses angiogenesis, and inhibits processes associated with metastasis, such as EMT and matrix metalloproteinase activity. Numerous randomized clinical trials and meta-analyses have shown that the use of PSK in combination with chemotherapy may prolong survival and reduce the risk of recurrence in gastrointestinal cancers. At the same time, the preparation is characterized by relatively low toxicity and may reduce the immunosuppression caused by chemotherapy or radiotherapy. Despite many years of research, the mechanisms of action of PSK remain incompletely understood. They depend on both the state of the patient’s immune system and the properties of the tumor microenvironment [40].

**Table 4:** Selected bioactive products derived from *Trametes versicolor*: IC50/GI50 values against cancer cell lines. (* MTT assay; ** LDH assay). (↓ indicates a decrease; ↑ indicates an increase).

Product	Cell Line/Model	Cytotoxicity Parameters	Additional Biological Effects	Ref.
Aqueous extract	4T1 (breast cancer, mouse)	IC50 = 188.53 µg/mL ^(48 h)^ *	- Very weak antiproliferative activity against HCC (hepatocellular carcinoma), IEC-6 (normal rat intestinal epithelial cells) and Vero (normal green monkey kidney cells)	[41]
	DU145 (prostate cancer)	IC50 = 71.16 µg/mL ^(48 h)^ *		
Ethanol extract (fruiting body)	A375 (melanoma)	IC50 = 663.3 µg/mL ^(72 h)^ *		[36]
	SK-MEL-5 (melanoma)	IC50 = 358.4 µg/mL ^(72 h)^ *		
	Fibroblast (non-tumorigenic)	IC50 = 748.0 µg/mL ^(72 h)^ *		
Ethanol extract (mycelium)	SK-MEL-5 (melanoma)	IC50 = 88.6 µg/mL ^(72 h)^ *	- Induction of apoptosis - Induces PARP cleavage - Downregulation of p53 - Induction of autophagy (LC3-II ↑) - ↑ major histocompatibility complex (MHC) II presentation - ↑ programmed death-ligand 1 (PD-L1) - ↓ cell migration - Synergistic anticancer effect with paclitaxel	[36]
	A375 (melanoma)	IC50 = 114.5 µg/mL ^(72 h)^ *		
	Fibroblast (non-tumorigenic)	IC50 = 93.5 µg/mL ^(72 h)^ *		
Extract of *Trametes versicolor*	HUVEC (Human umbilical vein endothelial cells) (non-tumorigenic)	IC50 = 1185 µg/mL ^(24 h)^ * IC50 = 895 µg/mL ^(24 h)^ **	- LPS stimulation enhances the cytotoxicity of the extract *T. versicolor* - ↑ ROS, LPS stimulation enhances the effect - ↓ cell migration - ↓ LPS-Induced Release of IL-6, IL-8, and MMP-9	[42]
	MCF-7 (breast cancer)	IC50 = 984 µg/mL ^(48 h)^ * IC50 = 738 µg/mL ^(48 h)^ **		
Polysaccharide-rich extract	LoVo (colon cancer)	IC50 = 224.02 µg/mL ^(72 h)^ *	- Low toxicity to HT-29 colon cancer cell line - ↓ cell migration and invasion - ↑ expression of the E-cadherin - ↓ MMP-2 enzyme activity - ↑ 5-fluorouracil cell cytotoxicity	[43]
Musarin (protein isolated from polysaccharide peptide—PSP)	HCT15 (colon cancer)	3 μg/mL: 87% viable cells ^(96 h)^	- Apoptosis and necrosis not detected - Inhibits aggressive human colorectal cancer stem cell-like CD24^+^ CD44^+^ HT29 - EGFR tyrosine kinase inhibitor activity	[44]
	HT-29 (colon cancer)	3 μg/mL: 73% viable cells ^(96 h)^		
	T84 (colon cancer)	3 μg/mL: 61% viable cells ^(96 h)^		
5α,8α-epidioxyergosta-6,22-dien-3β-ol (Comp. **1**) (Figure 2)	SR (leukemia)	GI50 = 1.04 μM ^(48 h)^	- Screened against 58 human cancer cell lines and 58 gave positive results with a (MG_MID) of 20.06% (NCI)	[45]
	LOX IMVI (melanoma)	GI50 = 1.10 μM ^(48 h)^		
	T47D (breast cancer)	GI50 = 0.651 μM ^(48 h)^		

**Figure 2:**
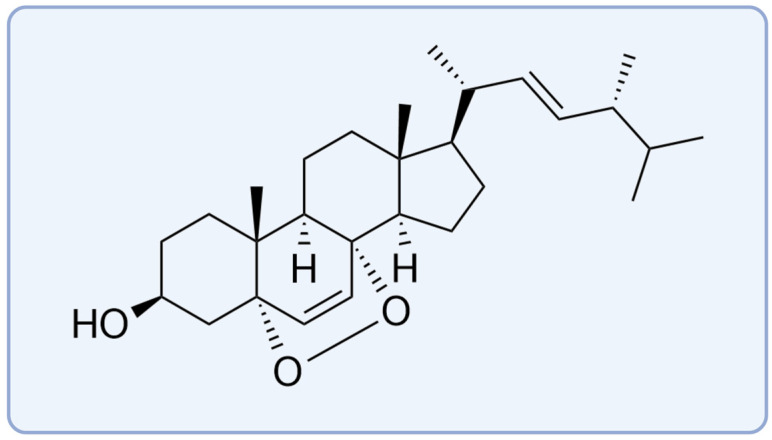
Chemical structure of 5α,8α-epidioxyergosta-6,22-dien-3β-ol (**Comp. 1**) isolated from *Trametes versicolor*. The characteristic 5α,8α-endoperoxide (peroxy) bridge, considered a key pharmacophore, has been associated with the compound’s anticancer activity [46].

### 3.5. Family Fomitopsidaceae—Fomitopsis betulina (Formerly Piptoporus betulinus)

**Figure 3:**
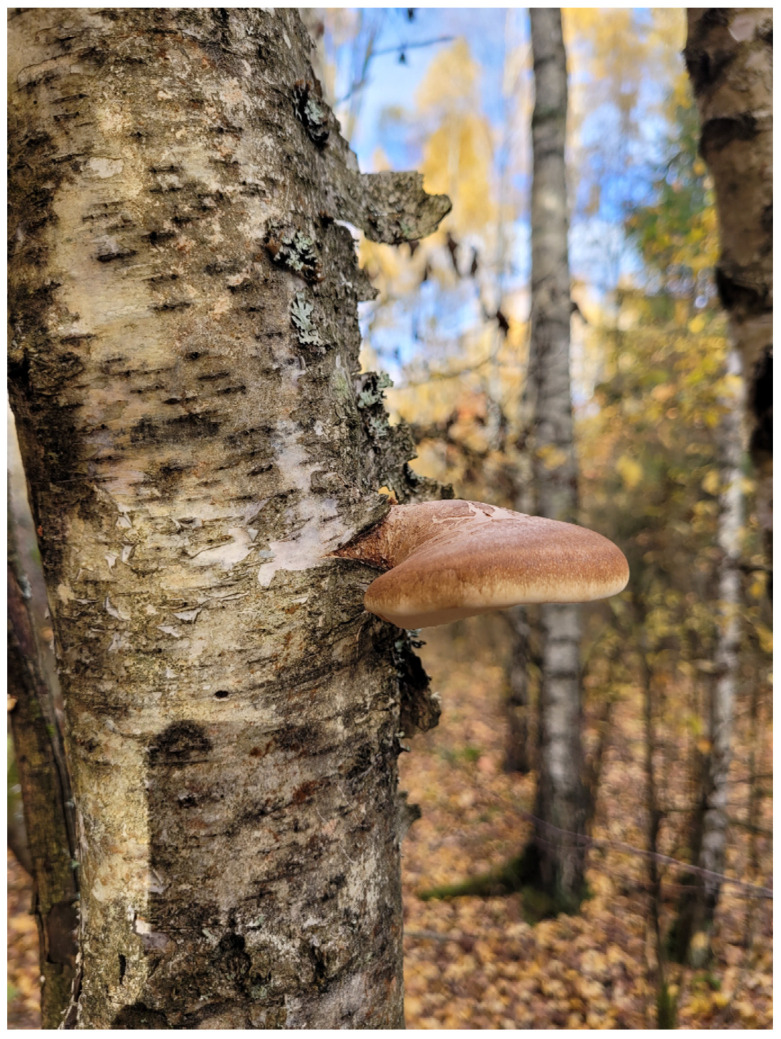
*Fomitopsis betulina* in its natural habitat in the Roztocze region, Poland.

*Fomitopsis betulina* (formerly *Piptoporus betulinus*), commonly known as birch polypore, birch bracket, or razor strop mushroom. It occurs on dead branches and trunks of various birch species in northern parts of Europe, North America, and Asia (Figure 3). It causes decay in old and weakened trees. Young fruiting bodies are edible, and dried fruiting bodies have traditionally been used in some Baltic countries to treat various types of cancer [47,48,49]. The fruiting bodies are annual, sessile to effused-reflexed, and tough to woody hard basidiocarps. Their pore surfaces range in color from white to pinkish. They grow singly or in small groups and are covered with a smooth, lacquered crust. Young fruiting bodies are white, turning grayish-brown as they age [50].

**Figure 4:**
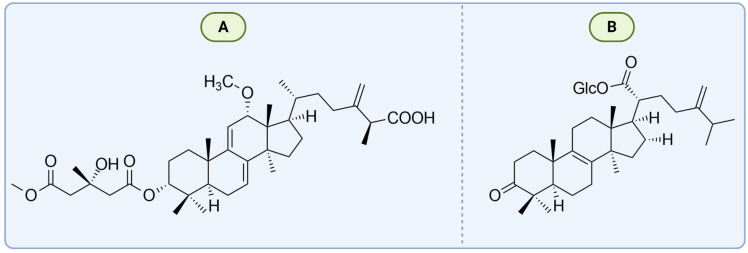
Chemical structures of piptolinic acid A (**A**) and fomitoside L (**B**), two lanostane-type triterpenoids isolated from *Fomitopsis betulina* with anticancer activity against leukemia cell lines.

In studies on the anticancer activity of *Fomitopsis betulina*, various extracts, fractions, polysaccharides, and terpenoid compounds were analyzed. Methanol extracts from cultivated mycelium and fruiting bodies were compared. They differed in chemical composition, particularly in the content of betulin and betulinic acid, which resulted in different biological effects [51]. An ethyl acetate fraction was isolated from the methanol extract of fruiting bodies. It showed activity against several cell lines while exhibiting low toxicity to normal cells [49]. Ethanol and aqueous extracts were also evaluated. In addition, extracts from fruiting bodies originating from artificial cultivation and natural sites were compared, as they differed in their potency [48]. In addition, the ethanol extract and betulin isolated from it were analyzed [52]. Aqueous extracts from fruiting bodies [53], and polysaccharide products such as gluco-oligosaccharides (α-(1 → 3)-GOS) were also analyzed [54]. Numerous lanostane-type triterpenoids were also identified, including the new compounds piptolinic acid A–E and fomitoside L–O. The active compounds included piptolinic acid A, fomitoside L (9) (Figure 4), fomitoside N (10), dehydropachymic acid (15), pachymic acid (16), fomitoside J (19), dehydrotumulose acid (22), and compound 29 (12α-hydroxy-3α-(3′-hydroxy-4′-methoxycarbonyl-3′-methylbutyryloxy)-24-methylanosta-8,24(31)-dien-26-oic acid) [47,55]. The results of studies on the anticancer activity of extracts and compounds isolated from *F. betulina* are summarized below (Table 5).

**Table 5:** Products obtained from *Fomitopsis betulina* with confirmed cytotoxic activity: % viable cells and IC50 values against cancer cell lines. (* MTT assay). (↓ indicates a decrease).

Product	Cell Line/Model	Cytotoxicity Parameters	Additional Biological Effects	Ref.
Methanol extract (mycelium)	DU145 (prostate cancer)	50 µg/mL: 27.28% viable cells ^(24 h)^ *	- Not cytotoxic to BJ (normal skin fibroblasts) and PNT-2 (normal prostate epithelial cells) - Anti-inflammatory activity	[51]
	A375 (melanoma)	50 µg/mL: 21.39% viable cells ^(24 h)^ *		
Methanol extract (fruiting body)	DU145 (prostate cancer)	50 µg/mL: 69.32% viable cells ^(24 h)^ *	- Not cytotoxic to BJ (normal skin fibroblasts) - Anti-inflammatory activity	
	A375 (melanoma)	50 µg/mL: 52.12% viable cells ^(24 h)^ *		
	PNT-2 (normal prostate cells)	50 µg/mL: 5.21% viable cells ^(24 h)^ *		
Ethyl acetate fraction from methanol extract	HT-29 (colon cancer)	50 µg/mL: ~40% viable cells ^(96 h)^ *	- Slight cytotoxic effect on normal cell lines, toxicity noticeable at a concentration of 200 µg/mL. - ↓ migration of C6 glioma cells - Morphological changes in A549 and C6	[49]
	A549 (lung cancer)	50 µg/mL: ~75% viable cells ^(96 h)^ *		
	C6 (glioma)	50 µg/mL: ~75% viable cells ^(96 h)^ *		
Ethanol extract	LS180 (colon cancer)	1–100 µg/mL: viable cells 90.07–48.05% ^(96 h)^ *	- Affected cell adhesion at 100 µg/mL	[56]
	A549 (lung cancer)	IC50 = 40.7 µg/mL ^(96 h)^ *	- Less active than ethanol extracts obtained from fruiting bodies grown in artificial culture (on birch sawdust) - Antimigratory activity	[48]
	HT-29 (colon cancer)	IC50 = 41.8 µg/mL ^(96 h)^ *		
	T47D (breast cancer)	IC50 = 48.1 µg/mL ^(96 h)^ *		
	A375 (melanoma)	10 µg/mL: ~20–30% viable cells ^(24 h)^	- Antioxidant activity - Selective action against melanoma cells (no active against human skin fibroblasts Hs27) - Caspase-3/7 activation only in cancer cells → apoptosis induction	[52]
	WM115 (melanoma)	10 µg/mL: ~50–70% viable cells ^(24 h)^		
Ethanol extracts (fruiting bodies artificially cultured)	A549 (lung cancer)	IC50 = 10.6–11.1 µg/mL ^(96 h)^ *	- Stronger antiproliferative and antimigratory activity for cultured extracts than wild-grown	[48]
	HT-29 (colon cancer)	IC50 = 15.4–27.6 µg/mL ^(96 h)^ *		
	T47D (breast cancer)	IC50 = 19.6–34.4 µg/mL ^(96 h)^ *		
Ether extract	LS180 (colon cancer)	1–100 µg/mL: viable cells 95.35–15.97% ^(96 h)^ *	- Affected cell adhesion at 100 µg/mL	[56]
Aqueous extracts	A549 (lung cancer)	IC50 = 6307 µg/mL ^(96 h)^ *	- Less active than aqueous extracts obtained from fruiting bodies grown in artificial culture (on birch sawdust) - Antimigratory activity	[48]
	HT-29 (colon cancer)	IC50 = 600 µg/mL ^(96 h)^ *		
	T47D (breast cancer)	IC50 = 210 µg/mL ^(96 h)^ *		
	LM-MEL-75 (melanoma)	IC50 = 2367.68 µg/mL ^(24 h)^ *	- Normal cells: IC50 > 20,000 µg/mL - Potential genotoxic/proapoptotic, cell cycle–targeting mechanism	[53]
	HT-29 (colon cancer)	IC50 = 2996.55 µg/mL ^(24 h)^ *		
	WEHI-164 (fibrosarcoma)	IC50 = 2175.99 µg/mL ^(24 h)^ *		
	A549 (lung cancer)	IC50 = 3256.86 µg/mL ^(24 h)^ *		
Aqueous extracts (fruiting bodies artificially cultured)	A549 (lung cancer)	IC50 = 1599–1819 µg/mL ^(96 h)^ *	- Stronger antiproliferative and antimigratory activity for cultured extracts than wild-grown	[48]
	HT-29 (colon cancer)	IC50 = 186–279 µg/mL ^(96 h)^ *		
	T47D (breast cancer)	IC50 = 61–124 µg/mL ^(96 h)^ *		
Betulin	A375 (melanoma)	0.05 µg/mL: viable cells ~40% (all tested lines)	- Stronger cytotoxic effect than crude extract - Likely due to higher concentration of single active compound vs. synergistic multi-component extract	[52]
	WM115 (melanoma)			
	Hs27 (skin fibroblasts) (non-tumorigenic)			
	HL-60 (acute promyelocytic leukemia)	IC50 = 61.90 µM ^(72 h)^ *	- Low selectivity index	[47]
	A549 (lung cancer)	IC50 = 93.92 µM ^(72 h)^ *		
	MRC-5 (normal lung fibroblasts) (non-tumorigenic)	IC50 = 57.94 µM ^(72 h)^ *		
Glucooligosaccharides (α-(1 → 3)-GOS)	LS180 (colon cancer)	IC50 = 19.55 µg/mL ^(96 h)^ *	- Selective proapoptotic activity against cancer cells - No toxicity to normal fibroblasts (normal mouse skin fibroblasts L929 and human skin fibroblasts HSF)	[54]
	SW-620 (colon cancer)	IC50 = 14.92 µg/mL ^(96 h)^ *		
	SW948 (colon cancer)	IC50 = 18.30 µg/mL ^(96 h)^ *		
	HT-29 (colon cancer)	IC50 = 17.06 µg/mL ^(96 h)^ *		
	CCD 841 CoTr (normal colon epithelial cells) (non-tumorigenic)	IC50 = 39.38 µg/mL ^(96 h)^ *		
Lanostane-type triterpenoids from methanolic extract	HL-60 (acute promyelocytic leukemia)	Piptolinic acid A: IC50 = 1.77 µM ^(24 h)^ * Other compounds: IC50 > 10 µM ^(24 h)^ *	- Structure–activity: methoxy groups at *C*-12 and *C*-5′ may enhance cytotoxic effects	[55]
	THP-1 (human acute monocytic leukemia)	Piptolinic acid A: IC50 = 8.21 µM ^(24 h)^ * Other compounds: IC50 > 10 µM ^(24 h)^ *		
Compounds isolated from chloroform–ethanol (2:1) extract	HL-60 (acute promyelocytic leukemia)	Compounds: **9**, **10**, **15**, **16**, **19**, **22**, **29**: IC50 = 10.90–37.01 µM ^(72 h)^ *	- Selectivity index (SI > 5) for compounds **15**, **16**, and **22** → selective activity vs. HL-60 over MRC-5 normal fibroblasts	[47]
	A549 (lung cancer)	Compounds: **9**, **10**, **15**, **16**, **19**, **22**, **29**: IC50 = 43.54–114.68 µM ^(72 h)^ *		
	MRC-5 (normal lung fibroblasts) (non-tumorigenic)	Compounds: **9**, **10**, **15**, **16**, **19**, **22**, **29**: IC50 = 35.45–115.43 µM ^(72 h)^ *		

### 3.6. Family Fomitopsidaceae—Fomitopsis pinicola

**Figure 5:**
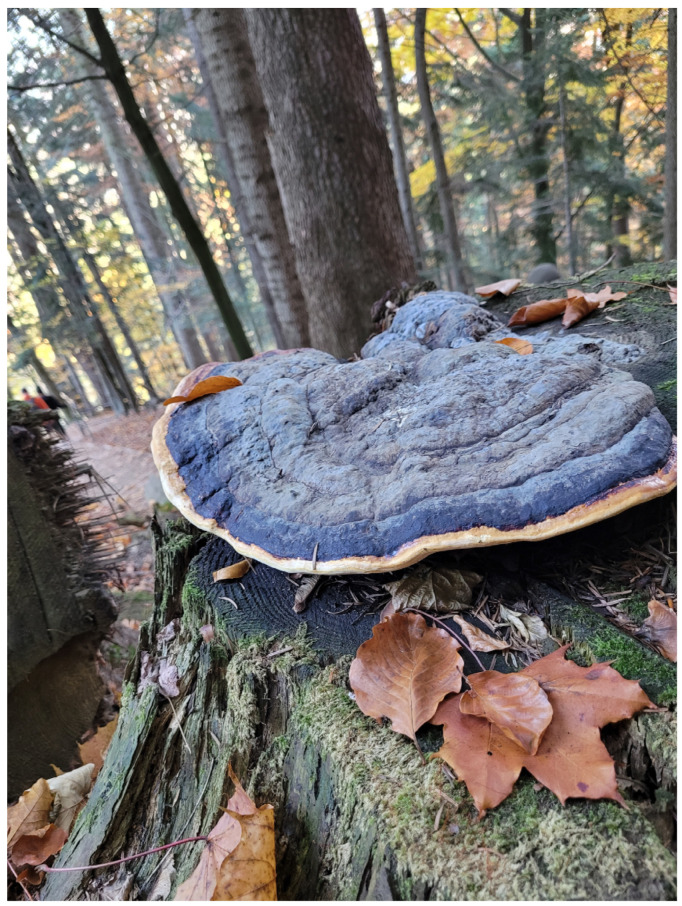
*Fomitopsis pinicola* in its natural habitat (Roztocze, Poland).

*Fomitopsis pinicola*, known as the brown-rot fungus, is a common wood-inhabiting species, occurring on coniferous and broad-leaved trees across Europe and Asia (Figure 5). The fruiting body has a fan-shaped form and a hard, woody texture. It can reach up to 40 cm in diameter and shows a glossy surface with reddish-brown shades. This species is both saprobic and parasitic. It causes brown rot in dead trees and heart rot in living ones. At the same time, it plays an important ecological role by decomposing dead trunks and participating in the circulation of nutrients in forest ecosystems. For centuries, it has been used in traditional Chinese and Korean medicine. It has been applied as an anti-inflammatory and antipyretic agent, as well as for the treatment of headaches, nausea, and liver diseases [57,58,59]. Reports on the anticancer activity of products isolated from this species are presented in the Table 6.

### 3.7. Family Laetiporaceae—Laetiporus sulphureus

*Laetiporus sulphureus* (Bull.) Murrill belongs to the *Polyporaceae* family. It is also known as “Sulphur Shelf’’ or “Chicken of the Woods”. It is common throughout the world, especially in Europe, Asia, and North America. It grows as a saprophyte on the bark of living deciduous and coniferous trees and on dead logs. It is most often found from May to October, mainly on oaks. It is an edible fungus with distinct yellow or orange shelf-like fruiting bodies. It has a characteristic mushroom-like aroma and a taste similar to chicken or crab. This resemblance is reflected in its common name. Due to its taste, it can be an alternative to a vegan diet [65,66]. *L. sulphureus* is a fungal species that possesses compounds with antimicrobial, antioxidant, and anticancer activities [67].

**Figure 6:**
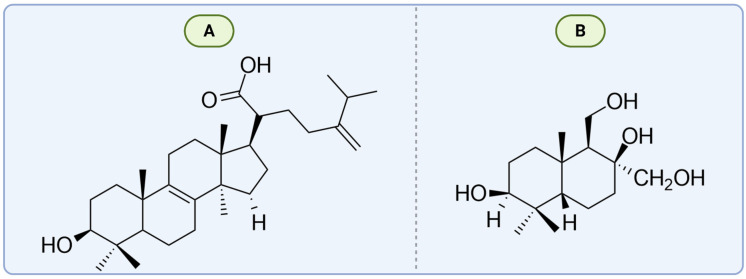
Chemical structures of eburicoic acid (**A**) and sulphureuin B (**B**), terpenoids isolated from *Laetiporus sulphureus* with anticancer activity.

Research into the anticancer activity of *Laetiporus sulphureus* has evaluated several extracts, protein compounds, and terpenoids. Among the tested extracts (water, ethanol, acetone, ethyl acetate, and chloroform), the ethanol extract showed the strongest activity, while the water and ethyl acetate extracts had moderate activity, and the acetone and chloroform extracts were the weakest [68]. A lectin was also isolated from the fruiting bodies of the fungus. In in vivo models, it showed strong anticancer and antiangiogenic activity without detectable toxicity [69]. Seven new driman-type sesquiterpenoids (sulphureuins B–H) and four known compounds were obtained from the ethyl acetate extract. Among them, eburicoic acid and sulphureuin B (Figure 6) showed the highest activity [70,71]. The results of studies on the anticancer activity of extracts, proteins, and terpenoids isolated from *L. sulphureus* are summarized below (Table 7).

### 3.8. Family Meripilaceae—Meripilus giganteus

*Meripilus giganteus* belongs to the family *Meripilaceae*. It is a wild, edible fungus that can be saprophytic or parasitic. It is found from summer to autumn on the trunks and roots of deciduous trees, mainly oaks and beeches. It is mainly found in the northern hemisphere—northern Europe, northern America and northern Asia. The upper part of the fruiting body is radially furrowed and has concentric streaks in shades of brown. Its surface is wrinkled and covered with scales. Young specimens have soft and tough flesh. With age, it becomes fibrous and white and blackens on contact or rubbing. Only young fruiting bodies are edible, due to the pericarp hardening with time. The species attracts attention for its known pharmacological properties, such as antioxidant, anticancer, and antimicrobial activity [72,73]. One of the first reports on the anticancer activity of this species came from France in 2003.

Methanol and ethanol extracts obtained from fruiting bodies and mycelium of *Meripilus giganteus* were analyzed in studies on its anticancer activity. They were tested against various cancer cell lines, including lung cancer, cervical cancer, colorectal cancer, and leukemia. Both cytotoxic and proapoptotic effects were demonstrated, as well as selectivity towards normal cells. The results concerning the activity of *M. giganteus* extracts are summarized below in Table 8.

## 4. Order *Hymenochaetales*

### Family Hymenochaetaceae—Inonotus hispidus

*Inonotus hispidus* is a parasitic fungus found mainly in China and Europe. It prefers deciduous trees such as *Fraxinus*, *Quercus*, *Sorbus*, and *Malus*. It is a popular edible and medicinal mushroom, known for its long history of use in traditional medicine. The fruiting bodies of *Inonotus hispidus* are annual, sessile, and woody in texture. In the early stages of growth, they are intensely yellow in color and edible. As they mature, they turn from light yellow to light brown, then dark brown, and at full maturity they become almost black, covered with characteristic hairy surfaces. Scientific reports indicate numerous health-promoting properties of this mushroom, including anticancer, antioxidant, antimicrobial, neurotrophic and neuroprotective, as well as immunomodulatory effects [75].

**Figure 7:**
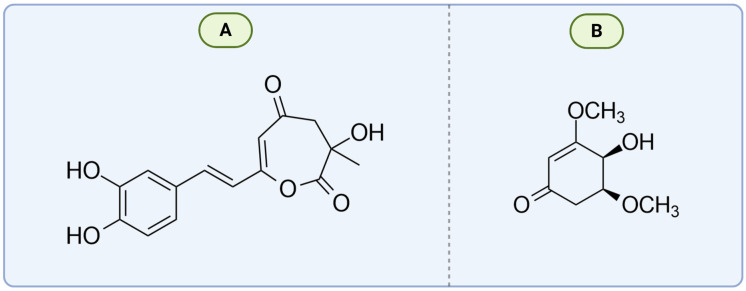
Chemical structures of compounds obtained from the methanol extract of *Inonotus hispidus*—inonotusin A (**A**) and HDE (**B**), fungal metabolites with anticancer activity in an in vitro model. The compounds differ in their characteristic functional groups, including a catechol moiety and lactone ring in inonotusin A and hydroxyl, methoxy, and carbonyl groups in HDE.

The anticancer activity of *Inonotus hispidus* was studied using a methanol extract of fruiting bodies. Phenolic compounds were isolated from the extract, including two new antioxidants, inonotusin A (Figure 7A,B), as well as the previously known hispidin and other phenolic derivatives [76]. The activity of the carbonyl compound (4S,5S)-4-hydroxy-3,5-dimethoxycyclohex-2-en-1-one (HDE) (Figure 7B) was also evaluated. The compound showed cytotoxicity against various cancer cell lines and the ability and induced apoptosis. Its anticancer properties with low systemic toxicity were also confirmed in an in vivo model [77]. The results for compounds isolated from *I. hispidus* are summarized below (Table 9).

## 5. Order *Auriculariales*

### Family Auriculariaceae—Auricularia auricula

*Auricularia auricula* (or *Auricularia auricula-judae*), commonly known as the wood ear mushroom or jelly ear fungus, is a species belonging to the order *Auriculariales*. It produces gelatinous, ear-shaped fruiting bodies with a brown to dark brown color. This species is a saprotrophic fungus that colonizes dead or decaying hardwood, contributing to lignocellulose degradation and nutrient cycling within forest ecosystems. The fruiting bodies exhibit desiccation tolerance, as they can rehydrate and regain their original morphology and reproductive capacity after prolonged drying. Recent molecular and phylogenetic studies have demonstrated that the traditionally recognized *Auricularia auricula-judae* represents a complex of closely related taxa with distinct genetic and biogeographical characteristics [78]. In addition to its ecological importance, *A. auricula* is widely consumed as an edible mushroom, particularly in Asian cuisine, and has attracted scientific interest due to its bioactive polysaccharides, which have shown potential antioxidant, immunomodulatory, and therapeutic properties [79]. Table 10 summarizes the reported anticancer activities of compounds isolated from this species.

## 6. Order *Agaricales*

### 6.1. Family Fistulinaceae—Fistulina hepatica

*Fistulina hepatica* belongs to the *Fistulinaceae* family and is also known as beefsteak fungus. An annual edible mushroom, this mushroom varies in color from red to brown. Its texture is meaty and juicy. It has a pleasant smell and a sour taste. Its common name refers to its appearance resembling raw meat. In the past it was consumed as a meat substitute. It is found in temperate and subtropical zones in deciduous forests. It grows as a saprophyte singly or in small groups, most often on oak or chestnut trees during summer and autumn [84,85]. To date, there are not many reports on biological activity.

To date, only one report has been published on the anticancer activity of *Fistulina hepatica*. The study investigated exopolysaccharides (EPS) isolated from submerged culture. In studies on rat glioma cells (C6), a 38% decrease in survival was observed after 24 h and a 45% decrease after 48 h at a concentration of 6 μg/mL [86].

### 6.2. Family Pleurotaceae—Pleurotus ostreatus

*Pleurotus ostreatus* is one of the most widely cultivated edible mushrooms in the world. It is characterized by its unique taste and high nutritional value. It is rich in protein, fiber, carbohydrates, vitamins, amino acids, and minerals while being low in fat. Due to its rapid growth and low technological and resource requirements, the oyster mushroom is readily available for commercial cultivation. It is also gaining increasing interest in the scientific community for its multidirectional biological properties. Among them are hypocholesterolemic, antiatherogenic, antioxidant, immune system-stimulating, and anticancer activities. These newly discovered biological activities have attracted considerable scientific interest. They also make the mushroom a potential ingredient in functional foods that support human health [87,88,89].

The extracts, nanoparticles, DNA, proteins, and polysaccharide fractions were analyzed in studies on the anticancer activity of *Pleurotus ostreatus*. Methanol and water extracts were obtained from the fruiting bodies, as well as a protein extract (PO). A proteoglycan fraction was isolated [87]. It was divided into four subfractions differing in the polysaccharide-to-protein ratio (crude, 11.1; I, 14.2; II, 26.4; III, 18.3). Several polysaccharide fractions were identified. These included HWS (hot water soluble) [90], the homogeneous polysaccharide POMP2 29 (kDa) [91], crude polysaccharides with three fractions (Mu1–Mu3) differing in carbohydrate content [92], and the water-extracted polysaccharide POP [89]. The study also evaluated AuNPs (gold nanoparticles) biosynthesized using the fungus and ChNPs (chitosan nanoparticles). These nanoparticles were tested both independently and in combination with the extract (PELChNPs) [93]. In addition, DNA isolated from fresh fruiting bodies was characterized [94]. Below is a summary of *P. ostreatus* products evaluated for anticancer activity (Table 11).

### 6.3. Family Physalacriaceae—Flammulina velutipes

*Flammulina velutipes*, also known as enokitake or golden needle mushroom, is an edible mushroom found throughout the world. It is prized for its high nutritional value and distinctive, rich flavor. It is particularly popular in Asian countries, where it is widely used in the food industry. It can be processed into a variety of products such as yogurts, beverages, noodles, meats or jellies. It is also used as a dietary supplement. The species is also known for its numerous pharmacological properties, including anticancer, cholesterol-lowering, antioxidant and immune-modulating effects [99,100].

A number of products with diverse chemical properties have been obtained from *Flammulina velutipes* (Table 12). These include extracts prepared using various solvents: acetone from fruiting bodies, water and methanol from caps, and water-alcohol from in vitro cultivated fruiting bodies, whose properties were further modified by gas plasma treatment.

In addition to solvent extracts, protein and polysaccharide compounds have also been identified. One of the best-characterized compounds is the glycoprotein FIP-fve (fungal immunomodulatory protein) [101]. It exhibits immunomodulatory properties and the ability to regulate the cell cycle. Among the carbohydrate fractions, FVP-1 (28 kDa), composed of glucose, galactose, mannose, and fucose, was isolated. FVP-2 (268 kDa), composed of glucose, mannose, galactose, xylose, and fucose, was also identified [102]. Three additional fractions, designated FVSP-1, FVSP-2, and FVSP-3, were isolated from the base of the fruiting body [99]. The crude polysaccharide FVP-C was isolated from the aqueous extract. After further purification, its main component, FVP-S1, was obtained, which is an acidic polysaccharide composed of glucuronic acid, xylose, and glucose [103].

**Table 12:** Products and extracts derived from *Flammulina velutipes*: IC50 values, % viability, and inhibitory rates against cancer cell lines. (* MTT assay). Abbreviations: FIP-fve, fungal immunomodulatory protein; FVP-1, polysaccharide fraction FVP-1; FVP-2, polysaccharide fraction FVP-2; FVSP-1, polysaccharide fraction FVSP-1; FVSP-2, polysaccharide fraction FVSP-2; FVSP-3, polysaccharide fraction FVSP-3; FVP-C, crude polysaccharide fraction; FVP-S1, acidic polysaccharide fraction. (↓ indicates a decrease; ↑ indicates an increase).

Product	Cell Line/Model	Cytotoxicity Parameters	Additional Biological Effects	Ref.
Acetone extract (fruiting bodies)	MCF-7 (breast cancer)	IC50 = 17.70 µg/mL ^(72 h)^ *	- Morphological changes consistent with apoptosis/cell death	[104]
	MDA-MB-231 (breast cancer)	IC50 = 114.50 µg/mL ^(72 h)^ *		
	MCF-10A (non-tumorigenic)	IC50 > 250 µg/mL ^(72 h)^ *		
Aqueous extract	BT-20 (breast cancer)	IC50 = 30 µg/mL ^(72 h)^ *	- Rapid apoptosis in MDA-MB-231 and MCF-7 - DNA fragmentation, strong apoptosis induction in MDA-MB-231 cell line	[105]
	MDA-MB-231 (breast cancer)	IC50 = 75 µg/mL ^(72 h)^ *		
	MCF-7 (breast cancer)	IC50 = 150 µg/mL ^(72 h)^ *		
Aqueous extract (caps)	MCF-7 (breast cancer)	IC50 = 14.42 µg/mL ^(72 h)^ *	- High antioxidant activity	[106]
	MDA-MB-231 (breast cancer)	IC50 = 151.57 µg/mL ^(72 h)^ *		
	MCF-10A (non-tumorigenic)	IC50 = 720.13 µg/mL ^(72 h)^ *		
Methanol extract (caps)	MCF-7 (breast cancer)	IC50 = 16.92 µg/mL ^(72 h)^ *	- High antioxidant activity	[106]
	MDA-MB-231 (breast cancer)	IC50 = 234.56 µg/mL ^(72 h)^ *		
	MCF-10A (non-tumorigenic)	IC50 = 942.43 µg/mL ^(72 h)^ *		
Gas plasma treated aqueous-alcohol extract (in vitro cultured)	MCF-7 (breast cancer)	Dose-dependent inhibition of proliferation after 24 h (no IC50 given)	- MCF-7: cell cycle arrest in G2/M phase - Activation of apoptosis pathway: ↑ expression of BAX, Caspase 3, p53 - ↑ oxidative stress, DNA damage observed - Strongest effects with 30 s plasma treatment (enhanced apoptosis markers and antitumor effect)	[107]
	MDA-MB-231 (breast cancer)			
	MCF-10A (non-tumorigenic)	No toxicity to MCF-10A		
FIP-fve	A549 (lung cancer)	15 µM: 60% viability ^(48 h)^ *	- ↑ p53 expression, ↓ p21 expression → G1 phase cell cycle arrest - Inhibition of A549 cell migration - ↓ filopodial fiber formation (via RacGAP1 silencing, affecting cytokinesis termination)	[101]
	MRC-5 (normal lung fibroblasts) (non-tumorigenic)	weaker effect than against A549		
FVP-1, FVP-2	A549 (lung cancer)	100 µg/mL: FVP-1—32.3% inhibitory rate ^(24 h)^ * 200 µg/mL: FVP-2—27.9% inhibitory rate ^(24 h)^ *		[102]
	BGC-823 (human gastric cancer)	200 µg/mL: FVP-1—78% inhibitory rate ^(24 h)^ * FVP-2—95% inhibitory rate ^(24 h)^ *		
FVSP-1, FVSP-2, FVSP-3	B16F10 (melanoma)	Inhibitory rate—50 µg/mL: FVSP-1—5.72% FVSP-2—7.30% FVSP-3—8.34%	- ↑ macrophage proliferation (141.8% at 50 µg/mL) - Indirect antiproliferative effect via cytokines from activated RAW264.7—approximately 2-fold increase in antiproliferative activity	[99]
	L929 (fibroblast, mouse) (non-tumorigenic)	Inhibitory rate—50 µg/mL: FVSP-1—1.45% FVSP-2—1.09% FVSP-3—3.30%		
FVP-C	A549 (lung cancer)	200 µg/mL: 17.08% viability	- ↓ migration - ↓ mitochondrial membrane potential - ↑ caspase-3, caspase-9 expression - ↑ Bax/Bcl-2 ratio → induction of apoptosis	[103]
FVP-S1		200 µg/mL: 25.07% viability		[103]

### 6.4. Family Schizophyllaceae—Schizophyllum commune

*Schizophyllum commune*, also known as “split-gill mushroom” or “white ginseng fungus,” is a widespread wood-decaying fungus, cultivated and appreciated for its unique flavor and aroma. Its immunomodulatory, anticancer, antimicrobial, antioxidant and anti-inflammatory activities are described in the scientific literature. The characteristic component of this species is schizophyllan—a β-glucan with wide biological activity, used in medicine, food and cosmetics. Other compounds present in *S. commune*, such as phenols, proteins, amino acids, polysaccharides and terpenoids, also show potential biological properties [108,109,110].

Schizophyllan (SPG) is a water-soluble homoglucan. Its backbone consists of glucose residues linked by β-(1 → 3) bonds, with single β-(1 → 6)-linked glucose side chains occurring on average every third residue. The compound is produced using strains of *Schizophyllum commune*, a fungus belonging to the basidiomycetes. In addition to its biological activity, schizophyllan is used in petroleum recovery processes, as a thickener in cosmetics and in the production of oxygen-impermeable films used in food preservation. Its biological activities include immunomodulatory properties, potential antiviral activity against HIV, and the ability to enhance the effects of vaccines and anticancer therapies. Studies have shown the efficacy of SPG against a variety of tumors in mice and rats, both alone and in combination with chemotherapy or radiation therapy. The observed effect depended on the presence of T lymphocytes. SPG stimulates the production of interleukins, interferon-γ, NK cell activity and lymphocyte proliferation, and enhances the effects of photodynamic therapy and irradiation. SPG derivatives show even stronger antitumor activity than the natural form. In clinical trials in Japan, the addition of SPG to standard treatment significantly prolonged survival and delayed disease recurrence in some cancers. The greatest benefit was observed in patients with high baseline levels of activated CD4+ and CD8+ lymphocytes. SPG is now approved for clinical use in Japan [111].

Various fractions obtained from *Schizophyllum commune* are a source of compounds with potential significance in cancer therapy (Table 13). Mycelium extracts obtained using chloroform and ethyl acetate were evaluated for cytotoxicity against selected cancer cell lines [112]. In addition, two polysaccharides with different structural and biological properties were isolated from the fruiting bodies of this species. The first of these, the highly branched glucomannan SCP-1 with a molecular weight of 15.1 kDa and good water solubility, consisted of fucose, glucosamine hydrochloride, galactose, glucose, and mannose [109]. The second compound, heteropolysaccharide SCFP with a molecular weight of 290.92 kDa, was dominated by mannose, galactose, and glucose. Both polysaccharides showed significant anticancer activity in in vitro models, and SCFP additionally also in an in vivo model [108].

**Table 13:** Products obtained from *Schizophyllum commune* with anticancer activity and their cytotoxicity parameters (IC50, % cell proliferation, and % tumor growth inhibition) against cancer cell lines. (* MTT assay). Abbreviations: SCP-1, polysaccharide fraction SCP-1; SCFP, heteropolysaccharide. (↓ indicates a decrease; ↑ indicates an increase).

Product	Cell Line/Model	Cytotoxicity Parameters	Additional Biological Effects	Ref.
Chloroform extract (mycelia)	HeLa (cervical cancer)	IC50 = 34.48 µg/mL ^(24 h)^ *	- Induction of apoptosis confirmed by AO/EB staining	[112]
	MCF-7 (breast cancer)	IC50 = 340.41 µg/mL ^(24 h)^ *		
	T47D (breast cancer)	IC50 = 5.44 µg/mL ^(24 h)^ *		
	WiDr (colon cancer)	IC50 = 19.44 µg/mL ^(24 h)^ *		
Ethyl acetate extract (mycelia)	HeLa (cervical cancer)	IC50 = 57.66 µg/mL ^(24 h)^ *		[112]
	MCF-7 (breast cancer)	IC50 = 420.48 µg/mL ^(24 h)^ *		
	T47D (breast cancer)	IC50 = 15.26 µg/mL ^(24 h)^ *		
	WiDr (colon cancer)	IC50 = 252.48 µg/mL ^(24 h)^ *		
SCP-1	A549 (lung cancer)	400 µg/mL: 58.38% cell proliferation ^(48 h)^	- Induced apoptosis in A549 cells - Shift in cell cycle distribution: G0/G1 ↓, S phase ↑ - ↓ Bcl-2/Bax ratio - ↓ Expression of mTOR, Akt, PI3K proteins	[109]
SCFP	U251 (human glioma)	IC50 = 3420 μg/mL ^(24 h)^ *	- No significant toxicity in vivo - Mechanism: modulation of PI3K/AKT signaling pathway - ↑ ARHI protein expression → ↓ proliferation, ↓ migration, ↑ apoptosis	[108]
	U87-MG (glioma)	IC50 = 2200 μg/mL ^(24 h)^ *		
	In vivo-Xenograft U251 model (human glioma), mice	47.39% tumor growth inhibition		

## 7. Order *Russulales*

### Family Bondarzewiaceae—Heterobasidion annosum

*Heterobasidion annosum* is one of the most harmful pathogens causing root and stem rot of coniferous trees in temperate zones around the world. This fungus belongs to a species complex that includes five intergroups: *Heterobasidion annosum sensu stricto*, *H. parviporum*, *H. abietinum*, *H. irregulare* and *H. occidentale*, which differ in their preference for hosts. In Europe, it most often attacks trees of the *Pinus*, *Picea*, *Abies* and *Juniperus* genus. Infection occurs through wounds and freshly cut trunks, and the fungus develops in the trunk and roots of both living and dead trees, causing white rot. The pathogen decomposes wood through the secretion of numerous enzymes. Identification is based, among other things, on the presence of fruiting bodies—irregularly shaped, with light-colored edges and a dark brown upper surface, reaching up to 40 cm in diameter. Small, brown, non-spore-forming papillae may be present on infected roots [113,114]. The first reports on the anticancer activity of the extract of this species are dated 2020.

A methanolic extract of *Heterobasidion annosum*, extracted from infected stems of *Pinus sylvestris* L. was analyzed in studies on the anticancer activity. The extract was evaluated in both in vitro and in vivo models, as well as in combination with 5-fluorouracil (5-FU) (Table 14) [113,115].

## 8. Summary of Anticancer Activity by Cancer Type

**Figure 8:**
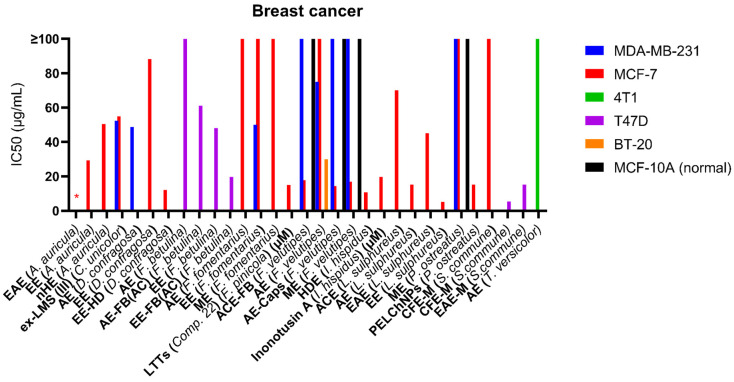
Anticancer activity of different extracts against the tested breast cancer cell lines. Values ≥ 100 are shown as ≥100. An asterisk (*) indicates values ≤ 1. Asterisks of different colors indicate the corresponding cell lines shown in the figure legend. Abbreviations: AE, aqueous extract; ACE, acetone extract; CFE, chloroform extract; CHE, cyclohexane extract; CME, crude methanolic extract; DCME, dichloromethane extract; EAE, ethyl acetate extract; EE, ethanol extract; ME, methanol extract; nHE, *n*-hexane extract; LTTs, lanostane-type triterpenoids; FB, fruiting bodies; FB(AC), artificially cultivated fruiting bodies; Caps, fruiting body caps; HD, hydrodistillation; M, mycelium; ex-LMS, low-molecular-weight fraction of secondary metabolites;; PELChNPs, *Pleurotus ostreatus* extract-loaded chitosan nanoparticles.

**Figure 9:**
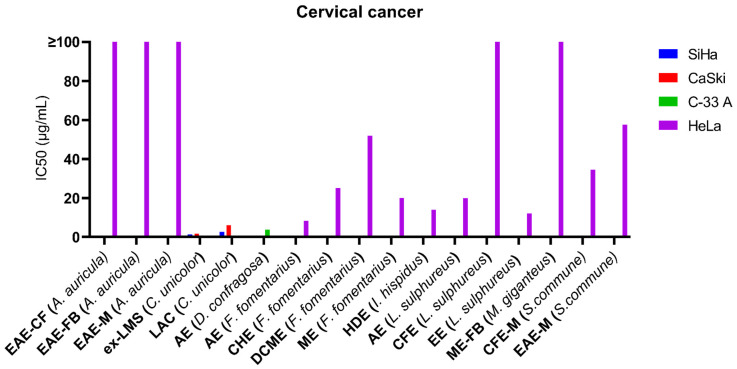
IC_50_ values of different extracts against the tested cervical cancer cell lines. Values ≥100 are shown as ≥100. Abbreviations: AE, aqueous extract; CHE, cyclohexane extract; CFE, chloroform extract; DCME, dichloromethane extract; EAE, ethyl acetate extract; EE, ethanol extract; ME, methanol extract; FB, fruiting bodies; CF, cultured fruiting bodies; M, mycelium; ex-LMS, low-molecular-weight fraction of secondary metabolites; LAC, laccase.

**Figure 10:**
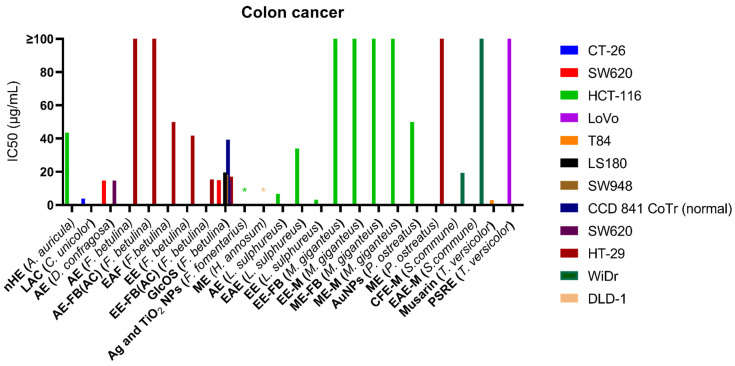
IC_50_ values of different extracts against the tested colon cancer cell lines. Values ≥ 100 are shown as ≥100. An asterisk (*) indicates values ≤ 1. Asterisks of different colors indicate the corresponding cell lines shown in the figure legend. Abbreviations: AE, aqueous extract; AuNPs, gold nanoparticles; CFE, chloroform extract; EAF, ethyl acetate fraction; EAE, ethyl acetate extract; EE, ethanol extract; GlcOS, glucooligosaccharides; LAC, laccase; ME, methanol extract; *n*HE, *n*-hexane extract; FB, fruiting bodies; FB(AC), artificially cultivated fruiting bodies; M, mycelium; NPs, nanoparticles; PSRE, polysaccharide-rich extract.

**Figure 11:**
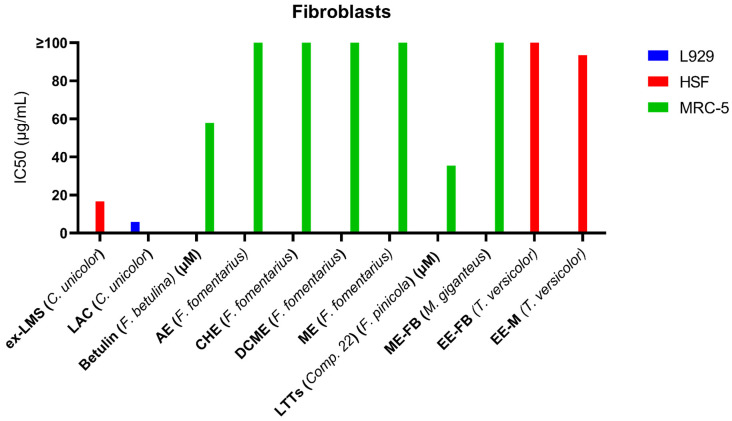
Cytotoxic activity of different extracts against the tested cell lines. Values ≥ 100 are shown as ≥100. Abbreviations: AE, aqueous extract; CHE, cyclohexane extract; DCME, dichloromethane extract; EE, ethanol extract; ME, methanol extract; FB, fruiting bodies; M, mycelium; ex-LMS, low-molecular-weight fraction of secondary metabolites; LAC, laccase; LTTs, lanostane-type triterpenoids.

**Figure 12:**
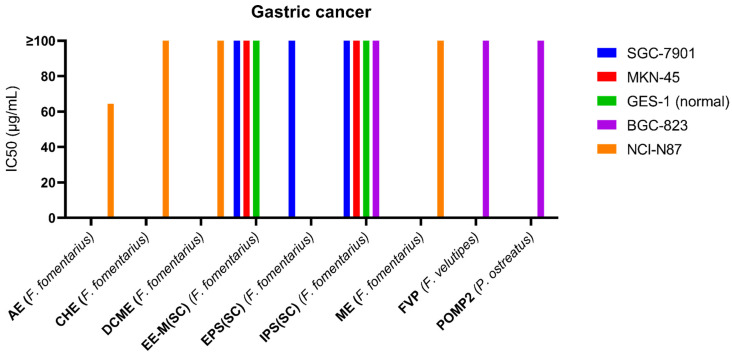
IC_50_ values of different extracts against the tested gastric cancer cell lines. Values ≥ 100 are shown as ≥100. Abbreviations: AE, aqueous extract; CHE, cyclohexane extract; DCME, dichloromethane extract; EE, ethanol extract; ME, methanol extract; EPS, exopolysaccharides; IPS, intracellular polysaccharides; FVP, polysaccharide fraction; POMP2, homogeneous polysaccharide; SC, submerged culture.

**Figure 13:**
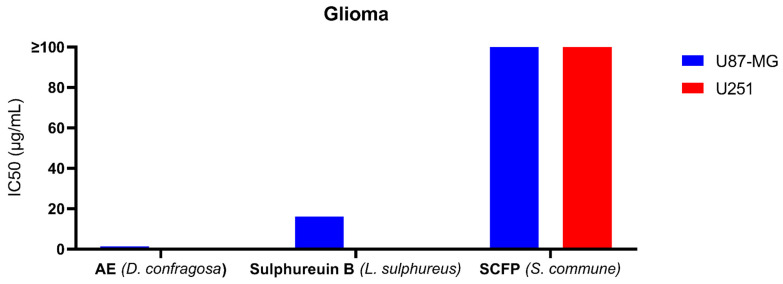
IC_50_ values of different extracts against the tested gliomas. Values ≥ 100 are shown as ≥100. Abbreviations: AE, aqueous extract; SCFP, heteropolysaccharide.

**Figure 14:**
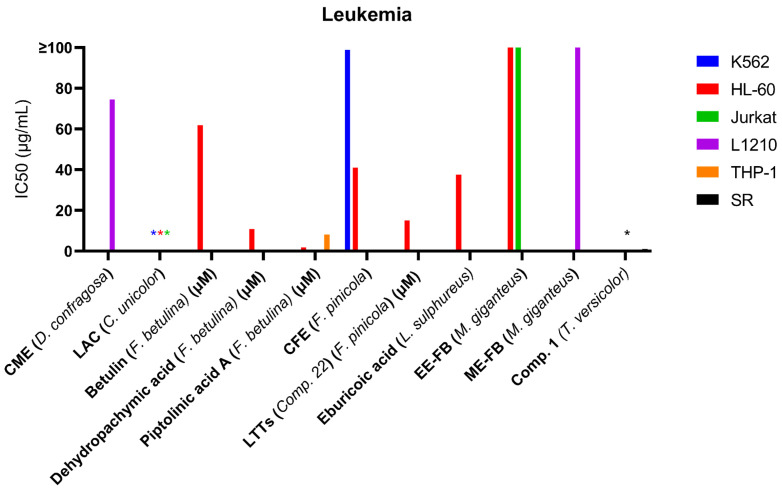
IC_50_ values of different extracts against the tested leukemias. Values ≥ 100 are shown as ≥100. An asterisk (*) indicates values ≤ 1. Asterisks of different colors indicate the corresponding cell lines shown in the figure legend. Abbreviations: CME, crude methanolic extract; CFE, chloroform extract; EE, ethanol extract; ME, methanol extract; FB, fruiting bodies; LAC, laccase; LTTs, lanostane-type triterpenoids.

**Figure 15:**
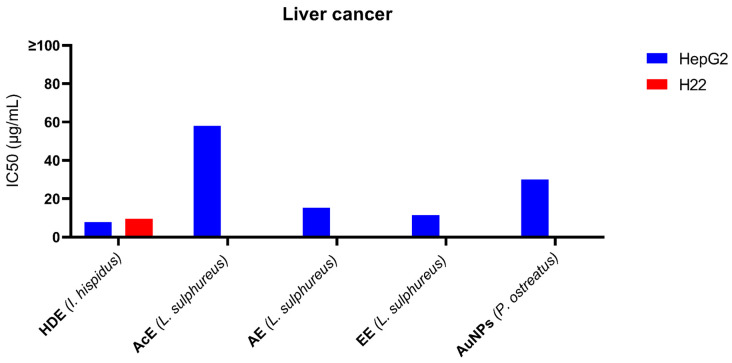
IC_50_ values of different extracts against the tested liver cancer cell lines. Values ≥ 100 are shown as ≥100. Abbreviations: AcE, acetone extract; AE, aqueous extract; EE, ethanol extract; AuNPs, gold nanoparticles.

**Figure 16:**
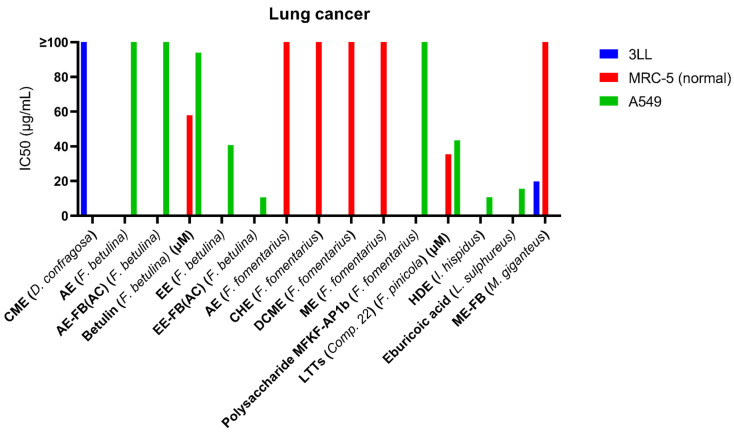
IC_50_ values of different extracts against the tested lung cancer cell lines. Values ≥ 100 are shown as ≥100. Abbreviations: AE, aqueous extract; CHE, cyclohexane extract; CME, crude methanolic extract; DCME, dichloromethane extract; EE, ethanol extract; ME, methanol extract; FB, fruiting bodies; FB(AC), artificially cultivated fruiting bodies; LTTs, lanostane-type triterpenoids.

**Figure 17:**
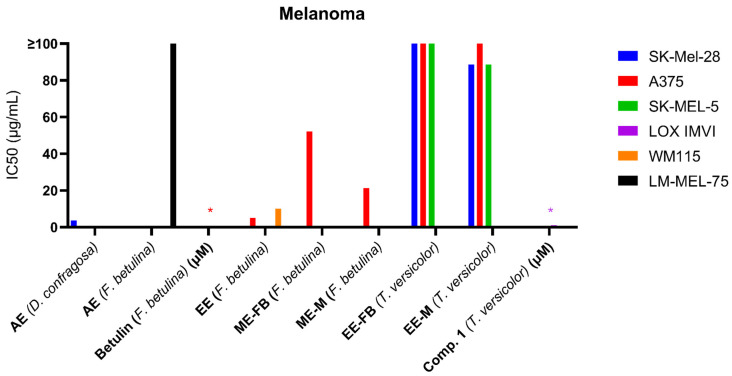
IC_50_ values of different extracts against the tested melanomas. Values ≥ 100 are shown as ≥100. An asterisk (*) indicates values ≤1. Asterisks of different colors indicate the corresponding cell lines shown in the figure legend. Abbreviations: AE, aqueous extract; EE, ethanol extract; ME, methanol extract; FB, fruiting bodies; M, mycelium.

**Figure 18:**
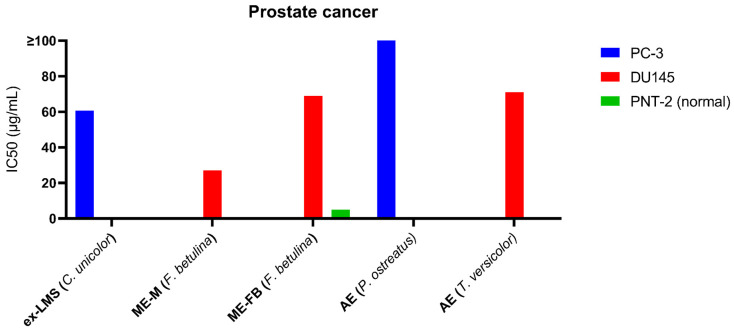
IC_50_ values of different extracts against the tested melanomas. Values ≥ 100 are shown as ≥100. Abbreviations: AE, aqueous extract; ME, methanol extract; FB, fruiting bodies; M, mycelium; ex-LMS, low-molecular-weight fraction of secondary metabolites.

In the previous sections, the anticancer activity of wood-inhabiting mushrooms was discussed according to individual species and their isolated bioactive compounds. However, this approach makes direct comparison of the effectiveness of specific extracts and compounds against particular cancer types difficult. Therefore, the available data are summarized in this section according to cancer type. This classification facilitates comparison of the anticancer activity of mushroom-derived extracts and isolated bioactive compounds across different cancer models and helps identify the mushroom species and constituents with the greatest therapeutic potential against specific malignancies. Figure 8, Figure 9, Figure 10, Figure 11, Figure 12, Figure 13, Figure 14, Figure 15, Figure 16, Figure 17 and Figure 18 provide an overview of the reported anticancer activities of mushroom-derived extracts and isolated bioactive compounds against individual cancer types, as well as their cytotoxicity toward normal fibroblasts.

## 9. Anticancer Applications of Wood-Based Fungi Biosynthetic Nanomaterials

Natural products are increasingly recognized as sustainable and biocompatible resources for the green synthesis of nanomaterials. Their extracts contain a wide range of bioactive compounds, including alkaloids, flavonoids, proteins, and polysaccharides, which can function as both reducing and stabilizing agents during nanoparticle synthesis. In this process, metal ions are reduced to their zero-valent state, while functional groups such as hydroxyl, amino, amide, and carboxyl groups contribute to nanoparticle stabilization, resulting in improved physicochemical properties and biocompatibility. Among natural resources, polysaccharides derived from wood-based fungi have attracted attention due to their intrinsic immunomodulatory and anticancer activities [116].

Recent advances in nanotechnology have expanded the potential applications of wood-based fungal polysaccharides in cancer therapy. *Ganoderma lucidum* polysaccharides have been employed in the synthesis of multifunctional tellurium nanorods exhibiting excellent photothermal conversion efficiency, which effectively inhibited tumor growth and metastasis, induced tumor cell apoptosis, and promoted dendritic cell maturation, thereby integrating photothermal therapy with chemo- and immunotherapy [117].

## 10. Molecular Mechanisms of Apoptosis Induced by Wood-Inhabiting Fungi

Apoptosis is one of the principal mechanisms responsible for the anti-cancer activity of bioactive compounds isolated from wood-inhabiting fungi. The available experimental evidence indicates that these compounds predominantly activate the intrinsic (mitochondrial) apoptotic pathway, characterized by disruption of mitochondrial membrane potential, cytochrome c release, activation of initiator caspase-9 and executioner caspase-3, together with modulation of Bcl-2 family proteins. Table 15 summarizes the molecular mechanisms of apoptosis induced by bioactive compounds isolated from wood-inhabiting fungi.

## 11. Emerging Molecular Targets Beyond Apoptosis

Epithelial–mesenchymal transition (EMT) is a fundamental process involved in tumor invasion, metastasis, and therapeutic resistance. It is characterized by the loss of epithelial markers, including E-cadherin, accompanied by increased expression of mesenchymal markers such as *N*-cadherin, vimentin, fibronectin, and EMT-related transcription factors. EMT is regulated by several signaling pathways, including TGF-β, Wnt/β-catenin, Notch, and PI3K/Akt [120].

Current evidence indicates that only a limited number of metabolites or extracts isolated from wood-inhabiting fungi have been experimentally investigated with respect to EMT regulation. A supercritical CO_2_ extract of *Ganoderma lucidum* spores was shown to suppress TGF-β1-induced EMT in human cholangiocarcinoma TFK-1 cells by restoring E-cadherin expression, reducing *N*-cadherin and fibronectin levels, inhibiting F-actin stress fiber formation, and consequently suppressing cell migration. These findings demonstrate that *G. lucidum* inhibits EMT predominantly through modulation of the TGF-β signaling axis rather than direct regulation of Wnt/β-catenin signaling [121].

**Figure 19:**
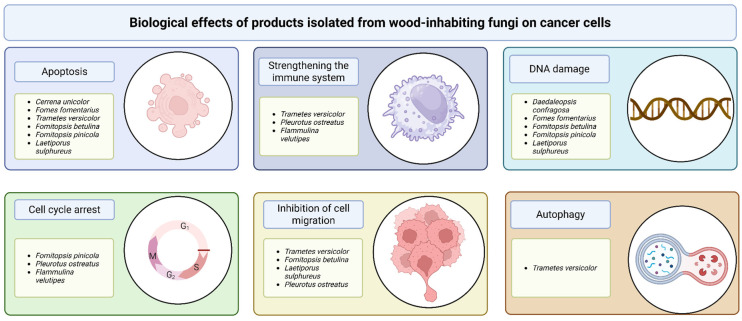
Biological effects of compounds isolated from wood-inhabiting fungi on cancer cells. Major anticancer activities reported for fungal metabolites and extracts, together with representative fungal species associated with each biological effect.

Evidence for direct modulation of Wnt/β-catenin signaling has been reported for *Phellinus linteus*. A protein-bound polysaccharide isolated from this mushroom inhibited proliferation, invasion, and angiogenesis of SW480 colon cancer cells by reducing β-catenin expression and its downstream targets, thereby attenuating Wnt/β-catenin signaling [122]. Similarly, *P. linteus* cultivated on germinated brown rice suppressed metastasis in a murine colon cancer model through inhibition of both NF-κB and Wnt/β-catenin pathways, accompanied by decreased expression of metastatic biomarkers [123]. In contrast, although compounds such as eburicoic acid, piptolinic acid, and inonotusin A exhibit cytotoxic and anti-proliferative activities, no original studies have demonstrated their direct regulation of EMT-associated molecular targets, including Wnt/β-catenin, Notch, or hTERT. Overall, the available evidence indicates that compounds isolated from wood-inhabiting fungi exert their anticancer activity primarily through the induction of apoptosis, cell cycle arrest, DNA damage, autophagy, inhibition of cell migration, and modulation of the immune response (Figure 19).

## 12. Conclusions and Future Perspectives

Wood-inhabiting fungi are an important source of biologically active compounds with anticancer potential. The bioactive compounds present in medicinal fungi may inhibit cancer cell proliferation and induce apoptosis, supporting their role as complementary therapeutic agents. Notably, PSK (Krestin) has been approved and used in clinical practice in countries such as Japan as an adjuvant treatment in cancer therapy, particularly in gastric and colorectal cancers. Although current laboratory and preclinical findings are highly promising, further large-scale clinical studies are still necessary to fully confirm their safety, efficacy, and mechanisms of action in humans.

Nevertheless, despite the encouraging results obtained from conventional in vitro assays and preclinical animal models, the clinical translation of fungal metabolites remains at an early stage. Future studies should focus on standardizing the cultivation and fermentation conditions of medicinal wood-inhabiting fungi to ensure the consistent production of bioactive compounds. Further investigation of structure–activity relationships and the development of novel delivery systems may improve the bioavailability and anti-cancer efficacy of fungal metabolites. Moreover, well-designed preclinical and clinical studies are needed to validate their therapeutic potential, optimize combination strategies with existing anti-cancer therapies, and facilitate their clinical translation.

## Figures and Tables

**Table 1:** Cytotoxicity parameters (EC50/IC50) and additional biological effects of products from Cerrena unicolor against cancer cell lines. (* MTT assay; ** BrdU assay; *** XTT assay). Abbreviations: LAC, laccase; ex-LMS, low-molecular-weight fraction of secondary metabolites.

Product	Cell Line/Model	Cytotoxicity Parameters	Additional Biological Effects	Ref.
LAC	Caov-3 (ovarian cancer)	100 µg/mL: ~35% survival ^(72 h)^ * ~50% proliferation ^(72 h)^ **	- Effects comparable to cisplatin but milder on normal cells (L929).	[19]
	NIH:OVCAR-3 (ovarian cancer)	100 µg/mL: ~50% survival ^(72 h)^ * ~90% proliferation ^(72 h)^ **		
	CT-26 (colon cancer, mouse)	IC50 = 3.85 µg/mL ^(72 h)^ **	- Inhibition of cancer cell migration	[20]
	L929 (fibroblast, mouse) (non-tumorigenic)	IC50 = 5.89 µg/mL ^(72 h)^ **		
	K562 (chronic myelogenous leukemia)	IC50 = 0.8 µg/mL ^(24 h)^ ***; IC50 = 1.0 µg/mL ^(48 h)^ ***	- Apoptosis confirmed (SEM—Scanning Electron Microscopy, Annexin V/Propidium iodide)	[21]
	HL-60 (acute promyelocytic leukemia)	IC50 = 0.5 µg/mL ^(24 h)^ ***; IC50 = 0.9 µg/mL ^(48 h)^ ***		
	Jurkat (T-cell leukemia)	IC50 = 0.8 µg/mL ^(24 h)^ ***; IC50 = 0.4 µg/mL ^(48 h)^ ***		
	RPMI 8226 (multiple myeloma)	IC50 = 0.9 µg/mL ^(24 h)^ ***; IC50 = 1.1 µg/mL ^(48 h)^ ***		
	SiHa (cervical cancer)	EC50 = 10.4 µg/mL ^(24 h)^ *; IC50 = 2.6 µg/mL ^(24 h)^ **	- Selective activity toward tumor cells (low effect on normal HSF cells) - Potent antiviral factor	[22]
	CaSki (cervical cancer)	EC50 = 60.9 µg/mL ^(24 h)^ *; IC50 = 6.0 µg/mL ^(24 h)^ **		
ex-LMS	SiHa (cervical cancer)	EC50 = 1.2 µg/mL ^(24 h)^ *; IC50 = 1.3 µg/mL ^(24 h)^ **		[22]
	CaSki (cervical cancer)	EC50 = 2.3 µg/mL ^(24 h)^ *; IC50 = 1.8 µg/mL ^(24 h)^ **		
	HSF (human skin fibroblasts) (non-tumorigenic)	EC50 = 16.6 µg/mL ^(24 h)^ *		
	PC-3 (prostate cancer)	ex-LMS I: IC50 = 97.17 µg/mL ^(48 h)^ *; ex-LMS II: IC50 = 103.32 µg/mL ^(48 h)^ *; ex-LMS III: IC50 = 60.66 µg/mL ^(48 h)^ *	- No activity against normal HSF cells - Antioxidant and antibacterial activities	[23]
	MDA-MB-231 (breast cancer)	ex-LMS I: IC50 = 74.91 µg/mL ^(48 h)^ *; ex-LMS II: IC50 = 89.85 µg/mL ^(48 h)^ *; ex-LMS III: IC50 = 52.25 µg/mL ^(48 h)^ *		
	MCF-7 (breast cancer)	ex-LMS I: IC50 = 66.22 µg/mL ^(48 h)^ *; ex-LMS II: IC50 = 109.45 µg/mL ^(48 h)^ *; ex-LMS III: IC50 = 54.92 µg/mL ^(48 h)^ *		

**Table 2:** Products from *Daedaleopsis confragosa* with anticancer activity and their cytotoxicity parameters (IC50/LD50 against cancer cell lines. (* MTT assay). Abbreviations: DC, conventionally prepared ethanol extract; DCHD, hydrodistillation-treated ethanol extract.

Product	Cell Line/Model	Cytotoxicity Parameters	Additional Biological Effects	Ref.
Ethanol extract	MCF-7 (breast cancer)	DC: IC50 = 88.23 µg/mL ^(24 h)^ * DCHD: IC50 = 12.15 µg/mL ^(24 h)^ *	- Genotoxic effect against δ virus DNA (*Podoviridae*); - DC richer in phenolics (antioxidant potential)	[25]
Crude methanolic extract	L1210 (mouse lymphocytic leukemia)	IC50 = 74.5 µg/mL *		[26]
	3LL (Lewis lung cancer, mouse)	IC50 > 100 µg/mL *		
Aqueous extract (K-3 and K-4)	U87-MG (glioma)	LD50 = 1.3–7.3 µg/mL ^(72 h)^	- Impaired mitochondrial function, inhibition of proliferation - K-3 inhibited tumor growth by 50.7% in vivo (21 days) - low toxicity vs. HEF-15 (normal fibroblasts)	[27]
	*C*-33 A (cervical cancer)	LD50 = 3.7–47.3 µg/mL ^(72 h)^ *		
	SK-Mel-28 (melanoma)	LD50 = 3.7–58.0 µg/mL ^(72 h)^ *		
	MDA-MB-231 (breast cancer)	LD50 = 48.7–290.0 µg/mL ^(72 h)^ *		
	SW-620 (colon cancer)	LD50 = 14.6–>58.0 µg/mL ^(72 h)^ *		

**Table 3:** Extracts and derivatives from *Fomes fomentarius* with anticancer activity and their cytotoxicity parameters (IC50/%survival) against cancer cell lines. (* MTT assay; ** XTT assay). Abbreviations: AgNPs, silver nanoparticles; TiO_2_NPs, titanium dioxide nanoparticles; EPS, exopolysaccharides; SC, submerged culture; IPS, intracellular polysaccharides; MFKF-AP1b, polysaccharide MFKF-AP1b. (↓ indicates a decrease; ↑ indicates an increase).

Product	Cell Line/Model	Cytotoxicity Parameters	Additional Biological Effects	Ref.
Cyclohexane extract	HeLa (cervical cancer)	IC50 = 25.10 µg/mL ^(72 h)^ *	- Antimicrobial activity - Anti-*Helicobacter pylori* activity	[31]
	NCI-N87 (stomach cancer)	IC50 > 200 µg/mL ^(72 h)^ *		
	MRC-5 (normal lung fibroblasts) (non-tumorigenic)	IC50 > 200 µg/mL ^(72 h)^ *		
Dichloromethane extract	HeLa (cervical cancer)	IC50 = 51.87 µg/mL ^(72 h)^ *	- Antimicrobial activity - Anti-*Helicobacter pylori* activity	[31]
	NCI-N87 (stomach cancer)	IC50 > 200 µg/mL ^(72 h)^ *		
	MRC-5 (normal lung fibroblasts) (non-tumorigenic)	IC50 = 111.59 µg/mL ^(72 h)^ *		
Methanol extract	HeLa (cervical cancer)	IC50 = 20.10 µg/mL ^(72 h)^ *	- Antimicrobial activity - Anti-*Helicobacter pylori* activity	[31]
	NCI-N87 (stomach cancer)	IC50 = 184.56 µg/mL ^(72 h)^ *		
	MRC-5 (normal lung fibroblasts) (non-tumorigenic)	IC50 > 200 µg/mL ^(72 h)^ *		
	MCF-7/S (drug-sensitive breast cancer)	IC50 = 350 µg/mL ^(72 h)^ **	- Multiple drug-resistant (MDR) modulator (P-gp modulation) in resistant sublines	[29]
	MCF-7/Pac (paclitaxel-resistant)	IC50 = 2000 µg/mL ^(72 h)^ **		
	MCF-7/Vinc (vincristine-resistant)	IC50 = 1800 µg/mL ^(72 h)^ **		
Aqueous extract	HeLa (cervical cancer)	IC50 = 8.31 µg/mL ^(72 h)^ *	- Antimicrobial activity - Anti-*Helicobacter pylori* activity	[31]
	NCI-N87 (stomach cancer)	IC50 = 64.46 µg/mL ^(72 h)^ *		
	MRC-5 (normal lung fibroblasts) (non-tumorigenic)	IC50 > 200 µg/mL ^(72 h)^ *		
	MCF-7/S (drug-sensitive breast cancer)	IC50 = 760 µg/mL ^(72 h)^ **		[29]
	MCF-7/Pac (paclitaxel-resistant)	IC50 = 1110 µg/mL ^(72 h)^ **		
	MCF-7/Vinc (vincristine-resistant)	IC50 = 1340 µg/mL ^(72 h)^ **		
Ethanol extract	MCF-7/S (drug-sensitive breast cancer)	IC50 = 1080 µg/mL ^(72 h)^ **	- Multiple drug-resistant (MDR) modulator (P-gp modulation) in resistant sublines	[29]
	MCF-7/Pac (paclitaxel-resistant)	IC50 = 2830 µg/mL ^(72 h)^ **		
	MCF-7/Vinc (vincristine-resistant)	IC50 = 1080 µg/mL ^(72 h)^ **		
	MDA-MB-231 (breast cancer)	50 µg/mL: ~50% survival ^(72 h)^ *	- Cell cycle arrest in S and G2/M phases (↓ CDK2, cyclin A/E, SKP-2; ↑ p21); - Apoptosis induction (↓ Bcl-2, PARP, ↑ cleaved caspase-9/-3) - Inhibited migration (↓ MMP-9, ↓ Akt phosphorylation, ↑ E-cadherin);	[35]
Ethanol extract from mycelium (SC)	SGC-7901 (gastric cancer)	800 µg/mL: ~10% survival ^(48 h)^ *		[32]
	MKN-45 (gastric cancer)	800 µg/mL: ~20% survival ^(48 h)^ *		
	GES-1 (normal gastric epithelial cells)	800 µg/mL: ~50% survival ^(48 h)^ *		
AgNPs and TiO_2_NPs	HCT-116 (colon cancer)	strong cytotoxicity at concentrations below 0.5 µg/mL ^(48 h)^ *	- Induction of cell death (chromatin condensation, nuclear disintegration) - Antibacterial activity	[33]
EPS (SC)	SGC-7901 (gastric cancer	500 µg/mL: ~30% survival ^(96 h)^ * 250 µg/mL: non-toxic ^(96 h)^ *	- EPS enhanced sensitivity of SGC-7901 to doxorubicin (3-fold increase in growth inhibition at 24 h)	[28]
IPS (SC)	SGC-7901 (gastric cancer)	800 µg/mL: ~30% survival ^(48 h)^ *		[32]
	MKN-45 (gastric cancer)	800 µg/mL: ~40% survival ^(48 h)^ *		
	GES-1 (normal gastric epithelial cells)	800 µg/mL: ~70% survival ^(48 h)^ *		
MFKF-AP1b	A549 (lung cancer)	100 µg/mL: 59.22% survival ^(24 h)^ * 43.74% LDH release ^(24 h)^	- Apoptotic morphology (apoptotic bodies, detachment - DNA damage confirmed by comet assay	[34]

**Table 6:** Products and extracts derived from *Fomitopsis pinicola* with anticancer activity and their cytotoxicity parameters (% viability, IC50, and inhibition rate) against cancer cell lines. (* MTT assay; ** MTS assay). Abbreviations: TiO_2_, titanium dioxide; AgNPs, silver nanoparticles. (↓ indicates a decrease; ↑ indicates an increase).

Product	Cell Line/Model	Cytotoxicity Parameters	Additional Biological Effects	Ref.
Hot water extract	HeLa (cervical cancer), SK-Hep3B (liver cancer), HO-1 (melanoma), PLC/RF/5 (liver cancer), SNU354 (hepatocellular carcinoma), SNU185 (hepatocellular carcinoma)	1000 µg/mL: 60–100% viability ^(48 h)^ * (SNU 185—60%)	- Antioxidant activity	[60]
Methanol extract		1000 µg/mL: 20–50% viability ^(48 h)^ * (HeLa—20%)		[60]
Ethyl acetate extract	DLA (lymphoma)	IC50 =100 µg/mL ^(24 h)^ *	- ↓ angiogenesis - Apoptosis induction - Cell cycle arrest in G1 phase	[58]
	In vivo-DLA (lymphoma) xenograft	Tumor weight was 57.93% less compared to control group		
Whiskey extract	In vivo-PC-3 (prostate cancer) xenograft	No observable differences in tumor growth	- Probably low bioavailability of active ingredients	[61]
Chloroform extract	SW-480 (colorectal cancer)	IC50 = 190.28 µg/mL ^(48 h)^ *	- Antimigration activity - DNA damage - Cell cycle arrest in the G1 phase - Apoptosis induction - ↑ ROS generation	[62]
	SW-620 (colon cancer)	IC50 = 143.26 µg/mL ^(48 h)^ *		
	Caco-2 (colorectal adenocarcinoma)	IC50 = 371,5 µg/mL ^(72 h)^ *		
	S180 (sarcoma)	IC50 = 104.9 µg/mL ^(48 h)^ * IC50 = 36.21 µg/mL ^(72 h)^ *	- ↓ cell membrane integrity (S180 cell line—24 h) - Apoptosis induction (↓ MMP, ↑ cytochrome c release) - ↑ DNA fragmentation - Cell cycle arrest in the S phase (S180 cells)	[57]
	K562 (chronic myelogenous leukemia)	IC50 = 139.81 µg/mL ^(48 h)^ * IC50 = 98.8 µg/mL ^(72 h)^ *		
	HL-60 (acute promyelocytic leukemia)	IC50 = 45.1 µg/mL ^(48 h)^ * IC50 = 40.99 µg/mL ^(72 h)^ *		
	U937 (pro-monocytic cell line)	IC50 = 39.07 µg/mL ^(48 h)^ * IC50 = 34.85 µg/mL ^(72 h)^ *		
	SMMC-7721(liver cancer)	IC50 = 320.12 µg/mL ^(48 h)^ * IC50 = 246.20 µg/mL ^(72 h)^ *		
	Eca-109 (esophageal carcinoma)	IC50 = 209.78 µg/mL ^(48 h)^ * IC50 = 169.73 µg/mL ^(72 h)^ *		
	In vivo-S180 (sarcoma) xenograft	Inhibition rate: 47.7% compared to control		
TiO_2_ and AgNPs	HCT-116 (colon cancer)	0,5 µg/mL: both ~20% viability ^(48 h)^ *	- Antibacterial activity	[63]
Lanostane-type triterpenoids	HL-60 (acute promyelocytic leukemia)	Comp. **9**: IC50 = 15.48–> 40 μM ^(48 h)^ ** Comp. **17**: IC50 = 13.62–17.52 μM ^(48 h)^ ** Comp. **18**: IC50 = 14.67–28.51 μM ^(48 h)^ ** Comp. **22**: IC50 = 11.47–16.74 μM ^(48 h)^ **	- Apoptosis induction	[64]
	A549 (lung cancer)			
	SMMC-7721 (liver cancer)			
	MCF-7 (breast cancer)			
	SW-480 (colorectal cancer)			

**Table 7:** Anticancer products obtained from *Laetiporus sulphureus*: IC50 values against cancer cell lines. (* MTT assay).

Product	Cell Line/Model	Cytotoxicity Parameters	Additional Biological Effects	Ref.
Ethanol extract	HCT-116 (colon cancer)	IC_50_ = 3.1 µg/mL ^(24 h)^ *	- Significant antimigratory effect vs. colon and cervical cancer (linked to pro-oxidant effect, strongest in HCT-116) - No toxicity to mouse hepatocytes	[67,68]
	MCF-7 (breast cancer)	IC_50_ = 5.1 µg/mL ^(24 h)^ *		
	HepG2 (liver cancer)	IC50 = 11.5 µg/mL ^(24 h)^ *		
	HeLa (cervical cancer)	IC50 = 12.1 µg/mL ^(24 h)^ *		
Aqueous extract	HCT-116 (colon cancer)	IC50 = 6.7 µg/mL ^(24 h)^ *	- No toxicity to mouse hepatocytes	[68]
	MCF-7 (breast cancer)	IC50 = 15.3–19.9 µg/mL ^(24 h)^ *		
	HepG2 (liver cancer)			
	HeLa (cervical cancer)			
Ethyl acetate extract	HCT-116 (colon cancer)	IC50 = 34–55 µg/mL ^(24 h)^ *	- No toxicity to mouse hepatocytes	[68]
	MCF-7 (breast cancer)			
	HepG2 (liver cancer)			
	HeLa (cervical cancer)			
Acetone extract	HCT-116 (colon cancer)	IC50 = 58–88 µg/mL ^(24 h)^ *		
	MCF-7 (breast cancer)			
	HepG2 (liver cancer)			
	HeLa (cervical cancer)			
Chloroform extract	HCT-116 (colon cancer)	No IC50 reported for tested cell lines (inactive)		
	MCF-7 (breast cancer)			
	HepG2 (liver cancer)			
	HeLa (cervical cancer)			
Lectin	In vivo-Danio rerio model with xenografts of HCT-116 (colon cancer) and B16-F10 (mouse melanoma)	Almost complete inhibition of colorectal cancer tumor growth (HCT-116)	- Potent antitumor activity in vivo - Anti-angiogenic efficacy > sunitinib-malate (clinical standard) - No toxicity even at high doses - In xenograft zebrafish model: inhibited tumor growth, metastasis, and tumor-induced angiogenesis - Strong antimigratory effect on endothelial cells → anti-angiogenic mechanism	[69]
Eburicoic acid (Figure 6A)	HL-60 (acute promyelocytic leukemia)	IC50 = 37.5 µg/mL ^(48 h)^ *		[71]
	SMMC-7721(liver cancer)	IC50 = 14.8 µg/mL ^(48 h)^ *		
	A549 (lung cancer)	IC50 = 15.6 µg/mL ^(48 h)^ *		
	SW-480 (Colorectal cancer)	IC50 = 36.1 µg/mL ^(48 h)^ *		
Sulphureuin B (Figure 6B)	U87-MG (glioma)	IC50 = 16 µg/mL ^(24 h)^ *	- Induced apoptosis with nuclear fragmentation, chromatin condensation, vacuolization - Apoptosis via 3 pathways: ER stress, mitochondrial, and receptor-mediated	[70]

**Table 8:** Methanol and ethanol extracts from *Meripilus giganteus* with anticancer activity and their cytotoxicity parameters (IC50, EC50, and % viable cells) against cancer cell lines (* MTT assay). (↓ indicates a decrease; ↑ indicates an increase).

Product	Cell Line/Model	Cytotoxicity Parameters	Additional Biological Effects	Ref.
Methanol extract (fruiting bodies)	L1210 (mouse lymphocytic leukemia)	IC50 > 100 µg/mL		[26]
	3LL (Lewis lung cancer, mouse)	IC50 = 19.8 µg/mL		
	HeLa (cervical cancer)	EC50 = 720 µg/mL ^(24 h)^ *, 410 µg/mL	- Induced apoptosis in HeLa cells - Antimigratory activity observed	[73]
	MRC-5 (normal lung fibroblasts) (non-tumorigenic)	EC50 > 1600 µg/mL ^(48 h)^ *		
Methanol extract (fruiting bodies and mycelium)	HCT-116 (colon cancer)	200 µg/mL: ~80% viable cells ^(48 h)^ *		[51]
	CCD 841 CoTr (normal colon epithelial cells) (non-tumorigenic)	no toxicity		
Ethanol extract (fruiting bodies)	Jurkat (T-cell leukemia)	IC50 = 385 µg/mL ^(24 h)^ *	- Induced apoptosis - ↑ BAX/BCL2 ratio - ↓ intracellular ROS levels - ↑ FAS gene expression - HL-60: G2/M arrest at 24 h → G0/G1 block at 48 h - Jurkat: no significant cell cycle effect	[72]
	HL-60 (acute promyelocytic leukemia)	IC50 = 461 µg/mL ^(24 h)^ *		
Ethanol extract (fruiting bodies and mycelium)	HCT-116 (colon cancer)	200 µg/mL: ~60% viable cells ^(48 h)^ *		[74]
	CCD 841 CoTr (normal colon epithelial cells) (non-tumorigenic)	no toxicity		

**Table 9:** Compounds isolated from *Inonotus hispidus* with anticancer activity and their IC50 values against cancer cell lines (* MTT assay). (↓ indicates a decrease; ↑ indicates an increase).

Product	Cell Line/Model	Cytotoxicity Parameters	Additional Biological Effects	Ref.
Inonotusin A	MCF-7 (breast cancer)	IC50 = 19.6 µM ^(120 h)^ *	- Antioxidant activity	[76]
HDE	HepG2 (liver cancer)	IC50 = 7.9 µg/mL ^(48 h)^ *	- Induced apoptosis in HepG2 cells - ↑ caspase-3 and caspase-8 activity (dose-dependent) - Fas receptor pathway activation: ↑ Fas, ↓ FasL expression - In vivo: inhibited tumor growth by up to 69% - No adverse effects on other organs (low systemic toxicity)	[77]
	MCF-7 (breast cancer)	IC50 = 10.7 µg/mL ^(48 h)^ *		
	HeLa (cervical cancer)	IC50 = 14.0 µg/mL ^(48 h)^ *		
	A549 (lung cancer)	IC50 = 10.7 µg/mL ^(48 h)^ *		
	H22 (liver)	IC50 = 9.5 µg/mL ^(48 h)^ *		

**Table 10:** Compounds isolated from *Auricularia auricula* with anticancer activity and their cytotoxicity parameters (IC50 and inhibition rate) against cancer cell lines (* MTT assay). (↓ indicates a decrease; ↑ indicates an increase).

Product	Cell Line/Model	Cytotoxicity Parameters	Additional Biological Effects	Ref.
*n*-Hexane extract	HCT-116 (colon cancer)	IC50 = 43.5 μg/mL ^(72 h)^ *	- α-amylase inhibition activity	[80]
	MCF-7 (breast cancer)	IC50 = 50.39 μg/mL ^(48 h)^ *		[81]
Ethanol extract	MCF-7 (breast cancer)	IC50 = 29.28 μg/mL ^(48 h)^ *		
	P388DI (macrophage cell line)	IC50 = 44.03 μg/mL ^(24 h)^ *		[82]
	S180 (sarcoma)	IC50 = 133.00 μg/mL ^(24 h)^ *		
Ethyl acetate extract	MCF-7 (breast cancer)	IC50 = 0.21 μg/mL ^(48 h)^ *	- Moderate antioxidant activity	[81]
	HeLa (cervical cancer)	Fruitbodies: IC50 = 538 μg/mL ^(24 h)^ * Mycelium: IC50 = 579 μg/mL ^(24 h)^ * Culture filtrate: IC50 = 2246 μg/mL ^(24 h)^ *		[66]
	P388DI (macrophage cell line)	IC50 = 143.80 μg/mL ^(24 h)^ *		[82]
	S180 (sarcoma)	IC50 = 108.90 μg/mL ^(24 h)^ *		
	HCT-116 (colon cancer)	IC50 = 45.3 μg/mL ^(72 h)^ *	- Weak antioxidant activity - α-amylase inhibition activity	[80]
Chloroform extract	HCT-116 (colon cancer)	IC50 = 64.4 μg/mL ^(72 h)^ *	- α-amylase inhibition activity	
Methanol extract	HCT-116 (colon cancer)	IC50 = 109.2 μg/mL ^(72 h)^ *	- Weak antioxidant activity	
Dichloromethane extract	P388DI (macrophage cell line)	IC50 = 38.32 μg/mL ^(24 h)^ *		[82]
	S180 (sarcoma)	IC50 = 94.20 μg/mL ^(24 h)^ *		
	In vivo-S180 (sarcoma) solid tumor in BALB/c mice	Reduced the tumor size in comparison to the control group (inhibition rate 49.23%)		
Butanol extract	P388DI (macrophage cell line)	IC50 = 94.62 μg/mL ^(24 h)^ *		
	S180 (sarcoma)	IC50 = 134.10 μg/mL ^(24 h)^ *		
Aqueous extract	P388DI (macrophage cell line)	IC50 = 28.19 μg/mL ^(24 h)^ *		
	S180 (sarcoma)	IC50 = 102.30 μg/mL ^(24 h)^ *		
Water soluble β-D-glucan	ACC (Acinar cell carcinoma)	0.005 and 0.05 μg/mL: inhibition ratios of 23.5% and 34.1%	- Induction of apoptosis (↑ Bax and ↓ Bcl-2 expression)	[83]
	In vivo-S180 (sarcoma) solid tumor in BALB/c mice	5, 20 and 40 mg/kg showed inhibition ratios of 16.4%, 39.1% and 37.7%.		

**Table 11:** Various products obtained from Pleurotus ostreatus with potential anticancer activity and their cytotoxicity parameters (IC50, % viable cells, and proliferation inhibition) against cancer cell lines. (* MTT assay) Abbreviations: AuNPs, gold nanoparticles; PELChNPs, Pleurotus ostreatus extract-loaded chitosan nanoparticles; HWS, hot water-soluble; POMP2, polysaccharide fraction POMP2. (↓ indicates a decrease; ↑ indicates an increase).

Product	Cell Line/Model	Cytotoxicity Parameters	Additional Biological Effects	Ref.
Methanol extract	MCF-7 (breast cancer)	1000 μg/mL: ~70% viable cells ^(24 h)^ *	- Morphological alterations in MCF-7 and HT-29 cells - Cell cycle arrest in G0/G1 phase - ↑ p53 and p21 expression in MCF-7 - Modulation of p21 expression in HT-29	[95]
	MDA-MB-231 (breast cancer)	1000 μg/mL: ~60% viable cells ^(24 h)^		
	HT-29 (colon cancer)	1000 μg/mL: ~20% viable cells ^(24 h)^		
	MCF-10A (non-tumorigenic)	1000 μg/mL: ~100% viable cells ^(24 h)^		
Aqueous extract	PC-3 (prostate cancer)	400 µg/mL: complete elimination of cells ^(24 h)^	- Morphological alterations after 2 h exposure - Induction of apoptosis (rapid and strong) - DNA damage induction - Comparable activity across 4 commercial oyster mushroom batches (U.S.)	[96]
Protein extract	SW-480 (Colorectal cancer)	10 µg/mL: ~40% viable cells ^(24 h)^; 500 µg/mL: ~2% viable cells ^(24 h)^;	- Likely mechanism: ROS generation (observed at 100 µg/mL) - ↓ GSH levels → oxidative stress response - ↓ mitochondrial membrane potential (ΔΨm) - DNA damage (↑ sub-G1 population)	[97]
	THP-1 (human acute monocytic leukemia)	500 µg/mL: ~3% viable cells ^(24 h)^;		
AuNPs	HepG2 (liver cancer)	25 µg/mL: 66.5% viable cells;	- Compared to commercial AuNPs: higher efficacy vs. HCT-116 and PC-3, lower vs. HepG2 - Synergistic antimicrobial effect with antibiotics (stronger than commercial AuNPs)	[98]
	HCT-116 (colon cancer)	25 µg/mL: 77.3% viable cells;		
	PC-3 (prostate cancer)	25 µg/mL: 85.4% viable cells;		
PELChNPs	MCF-7 (breast cancer)	IC50 = 15.25 µg/mL ^(24 h)^ *	- Stronger antitumor activity than extract alone - Apoptotic morphology and structural changes intensified with concentration	[93]
Fungal DNA	In vivo: mice with Ehrlich’s ascites carcinoma	Preventive treatment: survival prolonged to 80 days vs. 52 days in controls	- Immunomodulatory and biotherapeutic activity linked to unmethylated CpG motifs	[94]
Proteoglycan fractions (Crude, I, II, III)	S180 (sarcoma)	Fr. I: no activity; crude IC50 = 50 μg/mL; Fr. II–III: slightly higher activity	- Induced apoptosis in S180 ascites cells - Cell cycle arrest in sub-G0/G1 phase	[87]
	In vivo: S180 (sarcoma) ascites mice	Reduced tumor		
Aqueous polysaccharide extract (Fraction HWS)	HT-29 (colon cancer)	0.5% HWS (BSM 16:1): ↓ proliferation to 67.75% 0.5% HWS (BSM 32:1): ↓ proliferation to 93.75%	- Early apoptosis induction - ↑ Bax protein and cytochrome c expression → mitochondrial apoptosis pathway	[90]
	Fibroblasts (non-tumorigenic)	>0.05% HWS: no toxicity		
POMP2	BGC-823 (human gastric cancer)	400 μg/mL: 35.6% viable cell ^(72 h)^ *	- Inhibition of migration and colony formation	[91]
	In vivo-BGC-823 xenograft model mice (human gastric cancer)	Reduced tumor weight and volume at 100 and 200 mg/kg		
Crude polysaccharides and fractions (Mu1, Mu2, Mu3)	HepG2 (liver cancer)	No direct IC50 values reported Mu1 with stimulated NK cells has inhibitory effect: MCF-7 (57.2%), HepG2 (46.5%), and A549 (39.2%)	- No toxicity to peripheral blood cells of healthy donors - Stimulated NK cell proliferation (except Mu3) - Antitumor effect mediated by pre-activated NK cells (3 days activation + 5 days coculture) - ↑ TNF-α, IFN-γ, NO in NK-cancer cocultures	[92]
	MCF-7 (breast cancer)			
	A549 (lung cancer)			
Polysaccharide (POP)	EAC (Ehrlich Ascites Carcinoma)	200 μg/mL: ~50% viable cell ^(24 h)^ *; 1000 μg/mL: ~10% viable cell ^(72 h)^ *	- Confirmed antioxidant activity	[88]

**Table 14:** Methanol extract from *Heterobasidion annosum* with anticancer activity and its cytotoxicity parameter (% viability) against cancer cell lines. (* MTT assay). (↓ indicates a decrease; ↑ indicates an increase).

Product	Cell Line/Model	Cytotoxicity Parameters	Additional Biological Effects	Ref.
Methanol extract	DLD-1 (colon cancer)	1 µg/mL: ~50% viability ^(24 h)^ *	- ↓ Mitochondrial potential - ↑ Caspase-3 expression and nuclear translocation of caspase-3 and p53 - Combination with 5-FU: synergistic apoptotic response (↑ caspases-3, -8, and p53 in serum), no impairment of 5-FU effect	[113,115]
	In vivo-Xenograft DLD-1 model (colon cancer) (mice)	-Significant tumor volume reduction		

**Table 15:** Molecular mechanisms of apoptosis induced by bioactive compounds isolated from wood-inhabiting fungi. (↓ indicates a decrease; ↑ indicates an increase).

Compound/Product	Fungal Source	Apoptotic Mechanism	Proteins Affected	Reference
Ethanol extract	*Fomes fomentarius*	PARP cleavage	↓ Bcl-2, ↑ caspase-9/-3	[35]
Ethanol extract (mycelium)	*Trametes versicolor*	PARP cleavage, ↑ programmed death-ligand 1 (PD-L1)	↓ p53	[36]
Ethanol extract	*Fomitopsis betulina*		Caspase-3/7 activation	[52]
Chloroform extract	*Fomitopsis pinicola*	loss of mitochondrial membrane potential	↑ cytochrome c release	[57]
Sulphureuin B	*Laetiporus sulphureus*	ER stress, mitochondrial, and receptor-mediated		[70]
Ethanol extract (fruiting bodies)	*Meripilus giganteus*		↑ Bax and ↓ Bcl-2	[74]
HDE	*Inonotus hispidus*	extrinsic apoptosis pathway	↑ caspase-3 and caspase-8	[77]
Water soluble β-D-glucan	*Auricularia auricula*		↑ Bax and ↓ Bcl-2	[83]
Aqueous polysaccharide extract (Fraction HWS)	*Pleurotus ostreatus*	mitochondrial apoptosis pathway	↑ Bax protein and cytochrome c expression	[90]
Aqueous extract	*Flammulina velutipes*	DNA fragmentation		[105]
Gas plasma-treated aqueous-alcohol extract (in vitro cultured)	*Flammulina velutipes*	↑ oxidative stress, DNA damage observed	↑ expression of BAX, Caspase 3, p53	[107]
FVP-C and FVP-S1	*Flammulina velutipes*	↓ mitochondrial membrane potential	↑ caspase-3, caspase-9 expression; ↑ Bax/Bcl-2 ratio	[103]
SCP-1	*Schizophyllum commune*		↓ Bcl-2/Ba	[109]
Methanol extract	*Heterobasidion annosum*	↓ mitochondrial membrane potential	↑ Caspase-3 and p53	[113]
Ganoderic acid T	*Ganoderma lucidum*	Mitochondrial apoptosis, loss of mitochondrial membrane potential, cytochrome c release	↑ Bax, ↓ Bcl-2/Bax ratio, cytochrome c release, caspase-3 activation	[118]
Ganoderic acid Me	*Ganoderma lucidum*	Mitochondrial apoptosis	↑ Bax, ↓ Bcl-2/Bax ratio, cytochrome c release, ↑ caspase-3	[119]

## Data Availability

No new data were created or analyzed in this study. Data sharing is not applicable to this article.
